# Human Genome Replication Proceeds through Four Chromatin States

**DOI:** 10.1371/journal.pcbi.1003233

**Published:** 2013-10-10

**Authors:** Hanna Julienne, Azedine Zoufir, Benjamin Audit, Alain Arneodo

**Affiliations:** 1 Université de Lyon, Lyon, France; 2 Laboratoire de Physique, CNRS UMR 5672, Ecole Normale Supérieure de Lyon, Lyon, France; The Centre for Research and Technology, Hellas, Greece

## Abstract

Advances in genomic studies have led to significant progress in understanding the epigenetically controlled interplay between chromatin structure and nuclear functions. Epigenetic modifications were shown to play a key role in transcription regulation and genome activity during development and differentiation or in response to the environment. Paradoxically, the molecular mechanisms that regulate the initiation and the maintenance of the spatio-temporal replication program in higher eukaryotes, and in particular their links to epigenetic modifications, still remain elusive. By integrative analysis of the genome-wide distributions of thirteen epigenetic marks in the human cell line K562, at the 100 kb resolution of corresponding mean replication timing (MRT) data, we identify four major groups of chromatin marks with shared features. These states have different MRT, namely from early to late replicating, replication proceeds though a transcriptionally active euchromatin state (C1), a repressive type of chromatin (C2) associated with polycomb complexes, a silent state (C3) not enriched in any available marks, and a gene poor HP1-associated heterochromatin state (C4). When mapping these chromatin states inside the megabase-sized U-domains (U-shaped MRT profile) covering about 50% of the human genome, we reveal that the associated replication fork polarity gradient corresponds to a directional path across the four chromatin states, from C1 at U-domains borders followed by C2, C3 and C4 at centers. Analysis of the other genome half is consistent with early and late replication loci occurring in separate compartments, the former correspond to gene-rich, high-GC domains of intermingled chromatin states C1 and C2, whereas the latter correspond to gene-poor, low-GC domains of alternating chromatin states C3 and C4 or long C4 domains. This new segmentation sheds a new light on the epigenetic regulation of the spatio-temporal replication program in human and provides a framework for further studies in different cell types, in both health and disease.

## Author Summary

Previous studies revealed spatially coherent and biological-meaningful chromatin mark combinations in human cells. Here, we analyze thirteen epigenetic mark maps in the human cell line K562 at 100 kb resolution of MRT data. The complexity of epigenetic data is reduced to four chromatin states that display remarkable similarities with those reported in fly, worm and plants. These states have different MRT: (C1) is transcriptionally active, early replicating, enriched in CTCF; (C2) is Polycomb repressed, mid-S replicating; (C3) lacks of marks and replicates late and (C4) is a late-replicating gene-poor HP1 repressed heterochromatin state. When mapping these states inside the 876 replication U-domains of K562, the replication fork polarity gradient observed in these U-domains comes along with a remarkable epigenetic organization from C1 at U-domain borders to C2, C3 and ultimately C4 at centers. The remaining genome half displays early replicating, gene rich and high GC domains of intermingled C1 and C2 states segregating from late replicating, gene poor and low GC domains of concatenated C3 and/or C4 states. This constitutes the first evidence of epigenetic compartmentalization of the human genome into replication domains likely corresponding to autonomous units in the 3D chromatin architecture.

## Introduction

Understanding the role of chromatin structure and dynamics in the regulation of the nuclear functions including transcription and replication, is a major challenge of current research in genomics and epigenomics [1]–[7]. Since the initial sequencing of complete genomes and more than a decade ago of the human genome [8], the development of new techniques, in particular chromatin immunoprecipitation (ChIP) followed by massive parallel sequencing (ChIP-seq) [9], has enabled genome-wide analysis of many epigenetic modifications such as histone modifications, histone variant incorporation as well as of various DNA-binding proteins [6]. These techniques have been extensively applied to various eukaryotic genomes, from budding yeast [10], to plants [11], [12], worm [13], fly [14], [15], mouse [6], [16], [17] and human [6], [16]–[18], and have led to significant progress in our understanding of the chromatin landscape and its impact on gene regulation, replication origin specification and cell differentiation. Statistical analyses of these multivariate data sets have shown that this huge combinatorial complexity can be reduced to a surprisingly small number of predominant chromatin states with shared features namely four in *Arabidopsis thaliana* [19], five in *Caenorhabditis elegans* [20] and four [21] or five [22] in *Drosophila*. To our knowledge, no such a drastic dimensional reduction has been reported in mammalian organisms so far. The application of a multivariate Hidden Markov Model (HMM) [23] as well as the implementation of adapted pattern-finding algorithm [24], have confirmed that distinct epigenetic modifications often exist in well-defined combinations corresponding to different genomic elements like promoters, enhancers, exons, repeated sequences and/or to distinct modes of regulation of gene expression such as actualy transcribed, silenced and poised [23]–[26]. Some recent study [27] of chromatin mark maps across nine different human cell types has ultimately identified fifteen main chromatin types which is a relatively limited number of epigenetic states but probably not the optimal complexity reduction one may achieve in human and more generally in mammalian genomes. The analysis of a wide set of chromatin regulators that add, remove or bind histone modifications reported in Ref. [28], is a very encouraging step in this direction since six major groups or modules of chromatin regulators were shown to encompass the combinatorial complexity and to be associated with distinct genomic features and chromatin environments.

How epigenetic mechanisms and gene expression coordinate with DNA replication has been a long-standing question [1]–[6]. On the contrary to bacteria, yeast and viruses, the genomes of multi-cellular eukaryotes have no clear consensus DNA sequence element associated with replication initiation [29], [30]. Metazoan genomes duplicate through the coordinated activation of hundreds to thousands of replication origins that can be extremely site-specific or poorly defined with a broad site specification [31]. Indeed more origins are prepared in G1-phase than actively needed in S-phase [32]. Epigenetic mechanisms very likely take part in the spatial and temporal control of origin usage and efficiency in relation with gene expression [32]–[37]. For many years, elucidating the determinants that specify replication origins has been hampered by the very limited number of well established origins in human and more generally in mammals (a few tens versus a few ten thousands expected) [4], [32], [36], [38]. Only very recently, nascent DNA strands synthetized at origins were purified by various methods to map replication origins genome-wide in different eukaryotic organisms including *Arabidopsis thaliana* [39], Drosophila [40], mouse [40], [41] and human [18], [42]–[47]. Despite some inconsistency or poor concordance between certain of these studies [4], [48], some general trends have emerged confirming the correlation of origin specification with transcriptional organization [3], [4], [32]. The set of replication origins identified so far are strongly associated with annotated promoters and seem to be enriched in transcription factor binding sites [43], [44], [49] and in CpG islands [40], [41], [43]. However a significant proportion of origins do not seem to be controlled in the same way as gene transcription since they are in regions void of DNase-I-hypersensitive sites (DHSs) and of histone marks found at active promoters [3], [43]. Interestingly, it has been recently reported that replication origins may contain specific nucleotide sequences. Actually G-rich consensus motifs were shown to be associated with *Drosophila*, mouse and human origins [40], [47], [50]. These analysis have opened new perspectives towards the identification of mechanisms governing origin selection in mammals.

The recent blooming of genome-wide mean-replication timing (MRT) data in yeast [51], plants [52], worm [13], fly [53], [54], mouse [55]–[57] and human [58]–[61] has provided the opportunity to establish links between the spatio-temporal program of replication, transcription and chromatin structure [3]–[6], [62]. It is now well established that in higher eukaryotes, there is a significant correlation between GC-rich and gene-rich regions replicating early in the S-phase and in between AT-rich and gene poor regions replicating late [55], [58], [62]. But recent studies in mammals [56], [59] and *Drosophila* [63], have shown that during differentiation, some genes change expression without change in MRT and vice versa, thereby indicating that transcription is not the only controlling factor and that the epigenetically regulated chromatin structure is likely to be part of the game [3], [4], [6], [62]. In good agreement with previous studies in *Drosophila* [22], [63], genome-wide MRT profiles along mouse and human chromosomes in different cell lines reveal a correlation with epigenetic modifications [64]. Early replicating regions tend to be enriched in open chromatin marks H3K4me1, H3K4me2, H3K4me3, H3K36me3, H4K20me1 and H3K9 and H3K27 acetylation, whereas late replicating zones are mostly associated with H3K9me2 and to a lesser extent with H3K9me3 [56], [65]. Importantly, the dynamic changes in MRT observed during development come along with some subnuclear repositioning [56], [57], [65]–[69], early replicating euchromatin domains being generally at the interior of the nucleus whereas late replicating heterochromatic domains are more peripheral or near nucleoli [69]–[73]. Recent experimental studies of long-range chromatin interactions using chromosome conformation capture techniques [65], [74]–[76] have confirmed that 3D chromatin tertiary structure plays an important role in regulating replication timing. In particular, replicon size, which is dictated by the spacing between active origins, correlates with the length of chromatin loops [37], [77], [78]. But as questioned in Refs [76], [79], [80], the dichotomic picture proposed in early studies [65], [74], [75], where early and late replicating loci occur in separated compartments of open and closed chromatin respectively, is somehow too simple as previously questioned in a detailed analysis of replication fork velocity [79]. Identifying the epigenetic chromatin regulators of the spatio-temporal program of DNA replication will be a formidable step towards understanding the so-called replicon and replication foci [71], [81]–[84] in relation with their transcription counterpart, the transcription factories [71], .

Here we perform principal component analysis (PCA) [87] and classical clustering [88] on thirteen epigenetic mark maps in the K562 immature myeloid human cell line (the results of a similar analysis for the lymphoblastoid GM12878 cell line are reported in the Supplementary Data) at the resolution 100 kb of corresponding available MRT data, with the perspective of identifying the major types of chromatin states in relation with replication timing during S-phase. For this comparative analysis, we use as a guide the so-called replication U-domains that were shown to cover about half of the human genome for 7 different human cell types including ES, somatic and HeLa cells [80], [89]. In these megabase-sized domains, the MRT has a characteristic U-shape with early initiation zones at the borders and late replication at centers. Remarkably a significant overlap is observed between these replication U-domains in different cell types and also with the so-called skew N-domains [90]–[92], where the compositional skew profile accumulated in the germline can be decomposed into a replication-associated linearly decreasing component that shaped as a N [92]–[94] and a step-like transcription associated component that increases in magnitude with transcription and changes sign with gene orientation [92], [93], [95]–[97]. From the demonstration that the average replication fork polarity is directly reflected by both the compositional skew and the derivative of the MRT [80], [98], [99], it has been argued that the experimental observation of a MRT derivative that behaves as a N inside replication U-domains is the signature of a progressive inversion of replication fork polarity. These large-scale gradients of replication fork polarity in somatic and germline cells initiate from early initiation zones, also called “master” replication origins [100], [101], at U/N-domain borders that were found to be hypersensitive to DNaseI cleavage, to be associated with transcriptional activity and to present a significant enrichment in the insulator-binding proteins CTCF, the hallmarks of localized (∼200–300 kb) open chromatin structure [80], [101]. The analysis of chromatin interaction HiC [80] and 4C [76] data have revealed that these replication U/N-domains indeed correspond to high-order self-interacting chromatin units. The additional observation of a remarkable gene organization inside these domains with a significant enrichment of expressed genes nearby the bordering “master” replication origins [92], [102] sheds light on these U/N-domains as regions of highly coordinated regulation of transcription and replication by the chromatin structure. These structural and functional units are conserved in mouse [91], [92] and are robust to chromosome rearrangements [103] which indicates that they are likely to be a major determinant of genome evolution [104].

## Results/Discussion

### Combinatorial analysis of chromatin marks

**Figure 1:**
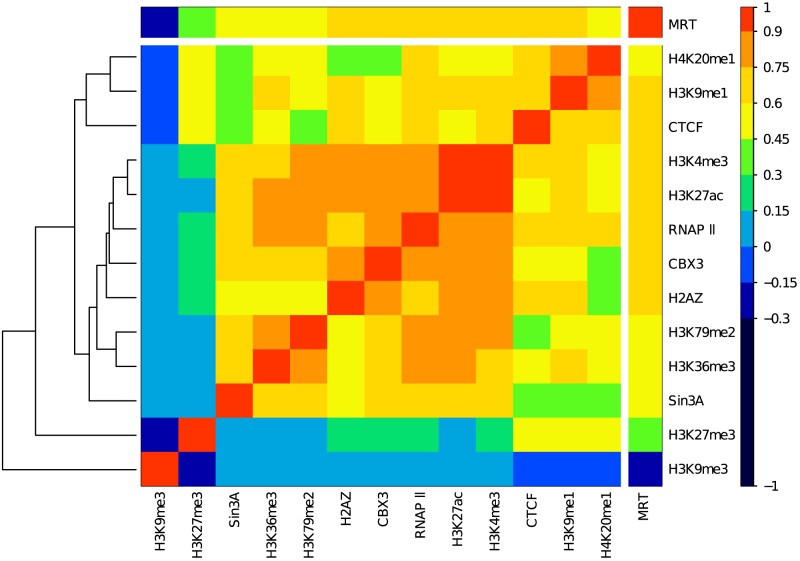
Spearman correlation matrix between epigenetics marks and mean replication timing (MRT). For each pair of variables we computed the Spearman correlation over all 100-overlapping windows with a valid score. Spearman correlation value is color coded using the color map shown on the right. A white line separates the MRT from epigenetics marks. Correlations with MRT (from late to early) are placed at the top and the right of the matrix. Lines for the thirteen epigenetic marks were reorganized by a hierarchical clustering using Spearman correlation distances (Equation 1) as illustrated by the dendrogram on the left of the graph. This ordering implies that highly correlated epigenetic marks are close to each other.

We investigated relationships between the genome-wide distributions of eight histone modifications, one histone variant and four DNA binding proteins in the immature myeloid human cell line K562 (Materials and Methods) at the 100 kb resolution of corresponding MRT data [61], [80]. As a first step, we computed the Spearman correlation coefficient of each mark with each other. We next represented the resulting matrix as a heat map after having reorganized rows and columns with a hierarchical clustering based on the Spearman correlation distance (Equation 1, Fig. 1). This preliminary analysis was very promising as regards to the possibility of reducing combinatorial complexity. All the epigenetic marks that are known to be involved in transcription positive regulation, namely H4K20me1, H3K9me1, H3K4me3, H3K27ac, RNAPII, CBX3, H2AZ, H3K79me2, H3K36me3, together with the transcription factors CTCF and Sin3A, form a block in the correlation matrix, meaning that they are all correlated with each other. The maximum correlation is actually obtained between the two active promoter marks H3K4me3 and H3K27ac. As suggested in Refs [27], [105], all these active marks are likely to occupy similar regions in the genome. In fact, two lines are clearly apart on the hierarchical clustering dendrogram (Fig. 1). They correspond to the repressive chromatin marks H3K27me3 and H3K9me3 that are respectively associated with the so-called facultative and constituve heterochromatins [105], [106]. These two marks are recognized by the chromodomains of polycomb (Pc) proteins and heterochromatin protein 1 (HP1), respectively, components of distinct gene silencing mechanisms which likely explains that they are strongly anticorrelated with each other. While H3K9me3 behaves quite independently with respect to most of the active chromatin marks, H3K27me3 correlates to some of them and especially to H4K20me1, H3K9me1 and CTCF. When further investigating the correlations between the thirteen considered chromatin marks and the MRT (Fig. 1), we found, consistently with previous works [56], [59], [61], [64], [65], a strong correlation for the transcriptionally active marks with early replication. Some moderate correlation was obtained for the Pc associated repressive marks H3K27me3 which contrasts with the significant anticorrelation observed for the constitutive heterochromatin mark H3K9me3 with late replication.

**Figure 2:**
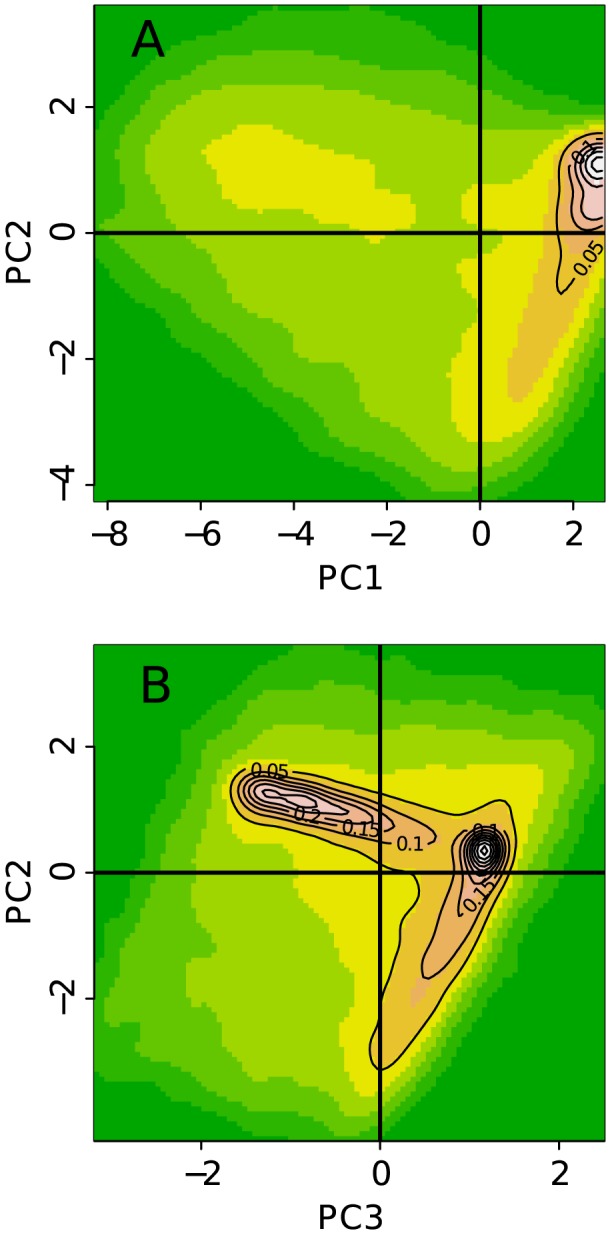
Principal Component Analysis (PCA). Two-dimensional (2D) projections of the data on (A) the plane defined by the first (PC1) and second (PC2) principal components, and (B) the plane defined by the second (PC2) and the third (PC3) principal components. The densities were computed by a kernel density estimation. The density values are indicated by a color (white: high density, yellow: moderate density, green: low density) and a contour plot.

**Figure 3:**
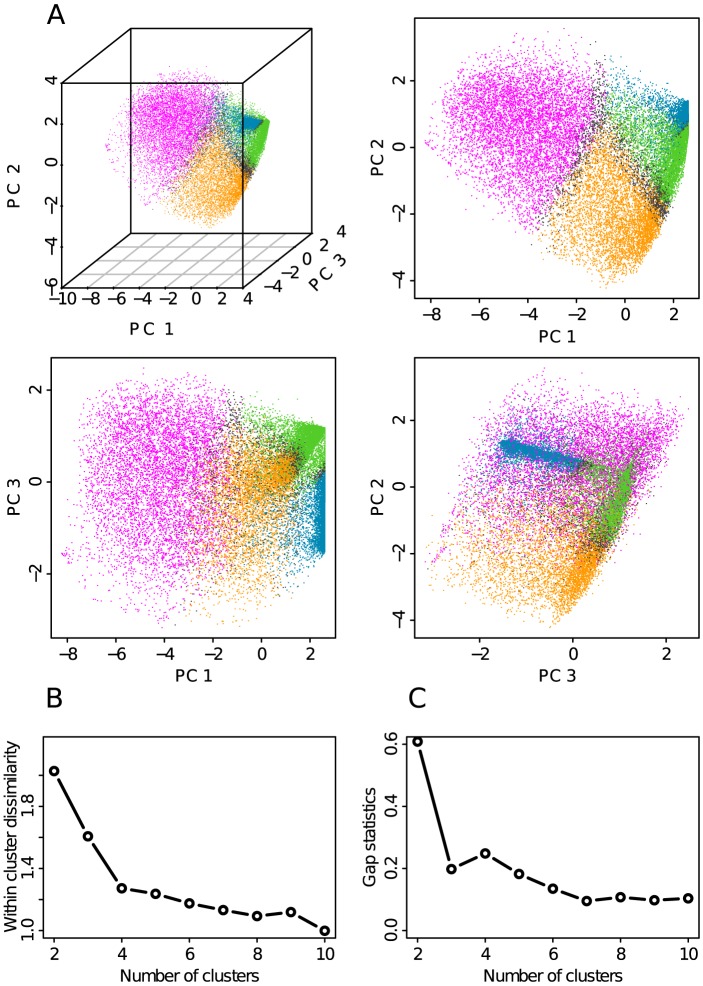
Defining the four prevalent chromatin states. (A) Scatterplot of the data points onto the first three principal components. Color dots indicate the four chromatin states as found by our clustering procedure (pink: transcriptionally active chromatin, orange: chromatin repressed by polycomb, green: silent unmarked chromatin, blue: HP1 heterochromatin). Points in dark grey are not classified in any chromatin state (see Materials and Methods). (B) Within-cluster sum of squares (Eq. 2) with respect to the number of clusters (see Materials and Methods). (C) Gap statistics (Eq. 4) with respect to the number of clusters (see Materials and Methods).

In a second step, to objectively identify the prevalent combinatorial patterns of the thirteen chromatin marks, we performed a PCA [107] to reduce the dimensionality of the data (Materials and Methods). We then concentrated on the first three principal components, which together account for 76% of the total data set variance (Supplementary Fig. S1). By projecting the 100 kb genomic loci on the (PC1, PC2) plane (Fig. 2A) and the (PC3, PC2) plane (Fig. 2B), we noticed that four areas contain most of the population. On the (PC1, PC2) plane, a large area of medium density comes out from a plane of much higher density. As viewed on the (PC3, PC2) plane, in this very dense plane, loci mainly lie along two straight lines with a very high density of loci concentrated at the intersection of these lines. This led us to use the Clara clustering algorithm [88], which is very similar to k-means, with the number of clusters fixed to four (Materials and Methods). When labeling each of the four main chromatin states with a color, we obtained four domains in the 3D scatter plot (Fig. 3A) that have common boundaries as evidenced on the three orthogonal projections on the planes (PC1, PC2) (Fig. 3B), (PC1, PC3) (Fig. 3C) and (PC3, PC2) (Fig. 3D). To improve the quality of our clustering procedure, we filtered out poorly clustered data points that are closer to another cluster than to the one they belong to (black dots in Fig. 3), where the distance between a data point and a cluster is defined as the mean of the distances of this point to all the points in the cluster. Removing those points is exactly equivalent as removing points with a negative silhouette [108] (Materials and Methods).

To determine the number of clusters, we used two statistical criteria (Materials and Methods). Four is the optimal choice according to the within-cluster sum of squares that clearly displays an elbow (abrupt slowing down of the decay) at the cluster number equal to four (Fig. 3B). The gap statistic [109] indicates that two or four clusters are good solutions (Fig. 3C). Our choice of four main chromatin states (Fig. 3A) can thus be seen as an attempt to test the limits of the classical dichotomic picture [65], [74], [75] of two chromatin states, one open (euchromatin) and another one closed (heterochromatin) (Supplementary Fig. S2A).

### Epigenetic content of the four prevalent chromatin states

**Figure 4:**
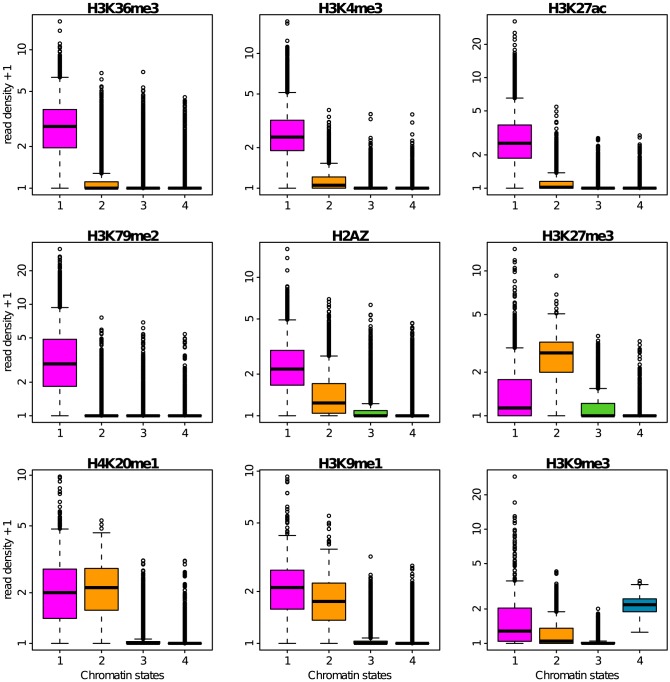
Repartition of histone marks in the four chromatin states. Boxplots of the decimal logarithm of histone mark ChiP-seq read density in 100 kb non-overlapping windows per chromatin state. Same color coding as in Fig. 3A.

**Figure 5:**
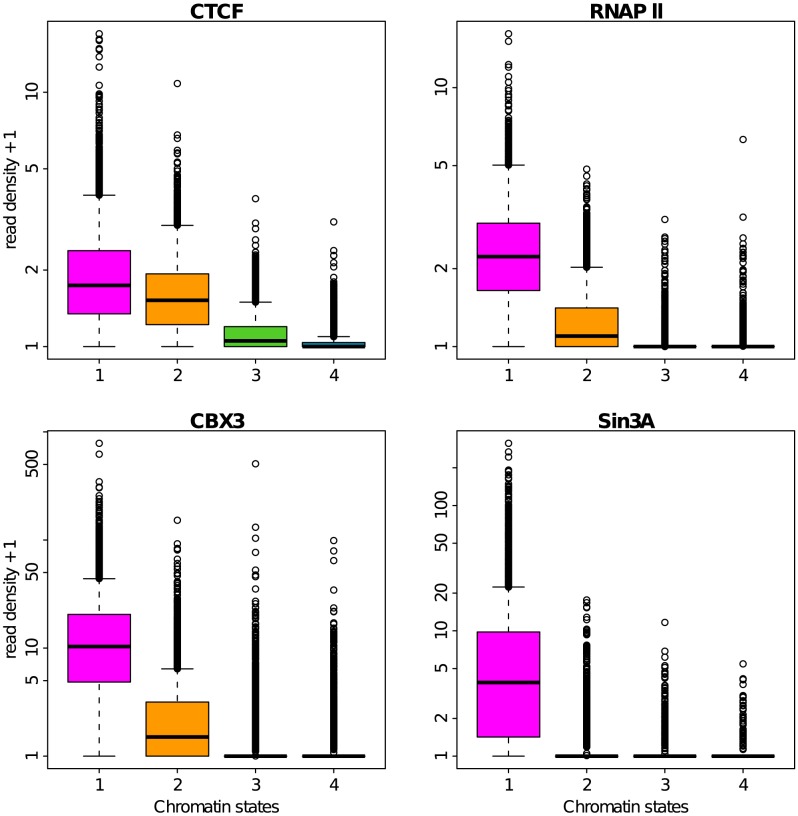
Repartition of transcription factors in the four chromatin states. Boxplots of the decimal logarithm of transcription factor ChiP-seq read density in 100 kb non-overlapping windows per chromatin state. Same color coding as in Fig. 3A.

The four prevalent chromatin states so identified and further labeled C1, C2, C3 and C4, were respectively found in 6572 (23.8%), 5312 (19.2%), 6603 (23.9%) and 6758 (24.4%) among the 27656 100 kb loci with a defined MRT (Materials and Methods). Indeed, we removed from the analysis the 2411 (8.7%) loci that were not properly classified in any chromatin state. More than 90% of the loci in C1 are associated (positive enrichment) with the histone modifications H3K36me3, H3K4me3, H3K27ac and H3K79me2, the hallmarks of transcriptionally active chromatin (Fig. 4) [2], [6], [105], as well as of the loci associated with RNA Polymerase II (Fig. 5) and the RPD3-interacting protein SIN3A (Fig. 5) as previously found in active euchromatin in *Drosophila* [22]. The majority of C1 loci are marked by H3k9me1 loci consistently with the observation of higher H3K9me1 levels in active promoters [105], and also contains the histone variant H2AZ whose binding level was shown to correlate with gene activity in human [105] (Fig. 4). C2 is notably associated with the histone modification H3K27me3 (Fig. 4), hence corresponds to a Polycomb repressed facultative heterochromatin state [105], [106]. Out of the four main chromatin states, C3 corresponds to 100 kb loci that are not enriched for any available marks. C3 can be compared to the “null” or “black” silent heterochromatin regions previously found in *Drosophila* [21], [22] and *Arabidopsis* [19] as covering a significant portion of the genome. C4 corresponds to the classic HP1-associated heterochromatin state with all of the 6603 C4 100-kb-loci containing the H3K9me3 mark and almost only that repressive mark (Fig. 4) [105], [106].

Methylation of H3K9 is well known to be implicated in heterochromatin formation and gene silencing [2]. The fact that H3K9me1 is found almost equally in C1 and C2 and not in C4 (Fig. 4), confirms that this epigenetic modification may also be associated with transcriptional activation [105]. H3K9me3 is found in all C4 100-kb-loci as the probable signature of its ability to anchor the heterochromatin protein HP1 at the origin of the establishment of heterochromatin. But H3K9me3 is not exclusively found in C4 loci; indeed 75% of C1 loci and 50% of C2 loci contain some H3K9me3 marks (Fig. 4). In the transcriptionally active state C1, H3K9me3 is present in combination with all active marks which might conduct in the anchoring of the 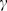 isoform of the HP1 protein [110]–[113], also called CBX3 (Fig. 5), which was recently shown to help the splicing of multiexonic genes [114], [115].

The insulator-binding protein CTCF is known to establish chromatin boundaries to prevent the spreading of heterochromatin into transcriptionally active regions [116], [117]. Consistent with the idea that CTCF-bound insulators prevent heterochromatin to invade genic regions, we found in good agreement with previous observation in *Drosophila* [21], [22] that CTCF is contained in C1 loci and to a slightly less extent in C2 loci (Fig. 5).

Despite the original association of H4K20 methylation with repressive chromatin [2], H4K20me1 was recently shown to strongly correlate with gene activation [105]. In particular when combined with H3K36me3 and H2BK5me1, this mark was found at highly expressed exons near human gene 5′-ends [118]. The high level of H4K20me1 found in C1 (Fig. 4) is quite consistent with these observations. However, we observed the same level of H4K20me1 in C2 which is silent. This suggests that this mark is not uniquely linked to transcription activation. Interestingly, recent works have confirmed that PR-Set7 involved in the deposition of H4K20me1 plays an important role in the control of replication origin firing in mammalian cells [119]–[121].

To assess the generality of the four prevalent chromatin states, we ran the same clustering procedure on the lymphoblastoid cell line GM12878 and on a third blood cell line (Monocyte CD14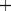, Monocd14ro1746). The same four main chromatin states emerged in the three cell lines (Supplementary Figs S7, S9, S10, S11). Hence the chromatin organization in four chromatin states is shared by at least several somatic human cell lines.

### Chromatin states are replicated at different times during S phase

**Figure 6:**
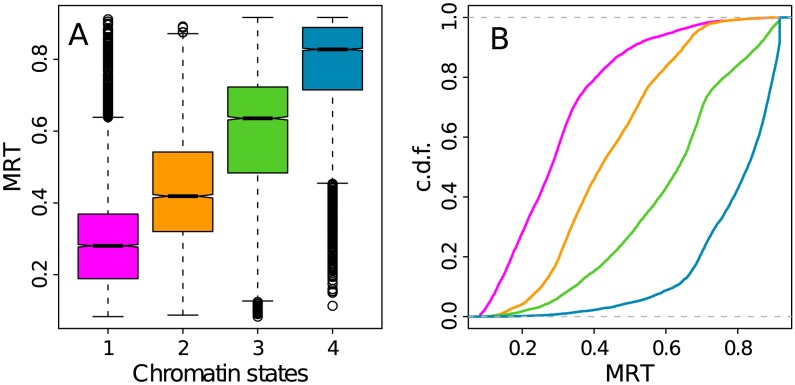
MRT in the four chromatin states. (A) Boxplots of MRT computed in 100 kb non-overlapping windows per chromatin state. (B) Empirical cumulative distribution function (c.d.f.) of MRT in the four chromatin states. Same color coding as in Fig. 3A.

This classification into four main chromatin states of the human genome shows strong similarities with those recently reported in *Arabidopsis* [19] and *Drosophila* [21], [22] suggesting the possible existence of some simple principles of epigenetic compartimentalization of eukaryotic genomes. However, what our study reveals with respect to previous works, is a strong correlation between these chromatin states and MRT (Fig. 6). C1, C2, C3 and C4 actually have significantly different MRT probability distribution functions (Fig. 6A) with a clear shift from early to late replicating as evidenced by the cumulative distribution functions (Fig. 6B). By applying a wilcoxon test to each pairs of chromatin states, we did verify that the p-value was infinitesimal. The transcriptionally active euchromatin state C1 replicates early in S phase consistent with previous analysis of open chromatin marks in human and mouse [56], [59], [61], [62], [64], [65]. The Pc-repressed facultative heterochromatin state C2 is replicated slightly later in mid-S phase which corroborates the recent finding of an association of H3K27me3 with mid-replicating chromosomal domains in human fibroblast [106]. This rather clear observation contrasts with previous contradictory results concerning the existence of high correlation between late replication and this repressive chromatin mark [65], [122]. The silent unmarked chromatin state C3 replicates later than C2 but before the HP1-associated heterochromatin state C4 that replicates very late almost at the end of S phase (Fig. 6). As previously reported in *Drosophila* [22], [63], these results confirm the existence of a strong link between epigenetic chromatin states and MRT in human. They further suggest that the epigenetically controlled chromatin structure has some impact on the normal progression of S-phase.

### Chromatin states are different functionally

To address the question of the gene content of these four prevalent chromatin states, we used a data set of 23818 genes that are spatially distinct (Materials and Methods). Some of these genes (3001) were not taken into account in our analysis because their promoter don't belong to any chromatin state. The mean density of the 20817 genes that belong to one of the four chromatin states is 8.24 promoters per Mb. The only chromatin state that is highly enriched in gene promoters is the early replicating euchromatin state C1 that harbours 62.0% of gene promoters even though it represents about 25% of the total genome coverage by the four chromatin states (Table 1 and 2). The mid S facultative heterochromatin state C2 also contains a non negligible percentage (19.6%) of gene promoters that indeed corresponds to a modest density 7.7 promoters/Mb as compared to 19.1 promoter/Mb found in C1. The late replicating unmarked and constitutive heterochromatin states C3 and C4 are genuinely gene deserts with very low gene densities 4.1 promoters/Mb and 1.8 promoter/Mb respectively. The mean gene length increases gradually from C1 to C4 going from 42.5 kb to 133.1 kb (Table 1). This discrepancy in gene length explains why the gene coverage decreases less abruptly than the promoter density, with C1 mainly genic (62.9%), C2 modestly genic (49.8%) and C3 (39.5%) and C4 (29.3%) mostly intergenic.

**Table 1:** Gene content in the four chromatin states.

Chromatin states	C1	C2	C3	C4
gene fraction (percent)	62.0	19.6	12.6	5.8
gene density per Mb	19.1	7.7	4.1	1.8
median gene length (kb)	19.0	19.0	17.8	26.1
mean gene length (kb)	42.5	59.4	83.5	133.1
gene coverage (percent)	62.9	49.8	39.5	29.3

For each chromatin state, the following information is given: (i) the fraction of genes in this state in percent of the total number of genes classified in the four chromatin states, (ii) the density of genes per Mb, (iii) the median gene length in kb, (iv) the mean gene length in kb and (v) the fraction of the chromatin state covered by genes in percent. The number of genes taken into account are 12904 genes in C1, 4089 in C2, 2625 in C3 and 1199 in C4.

**Table 2:** Domain organization of chromatin states.

Chromatin states	C1	C2	C3	C4	C1+C2	C3+C4
total length (Mb)	674.4	533.7	641.2	676.2	1367.9	1458.3
Number	2784	2612	2305	1021	1762	1804
mean(length)	275	228	325	718	779	808
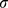 (length)	275.7	198.5	539.4	920.9	1175	1211.304
M0 mean	129	121	128	129		
M1 mean	242	204	284	667		
M1 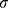	185.7	145.6	228.25	614.8		

The rows correspond to (i) the total length in Mb of each chromatin state, (ii) the number of each chromatin state domains, (iii) the mean length of each chromatin state domain in kb, (iv) the standard deviation of the length distribution for each chromatin state domain, (v) the expected length if each chromatin states were spatially independently distributed over 100-kb-loci, (vi) the expected length if 100-kb-loci chromatin state distributions are assumed to depend on their nearest neighbor and (vii) the length standard deviation given the same conditions as in (vi).

**Figure 7:**
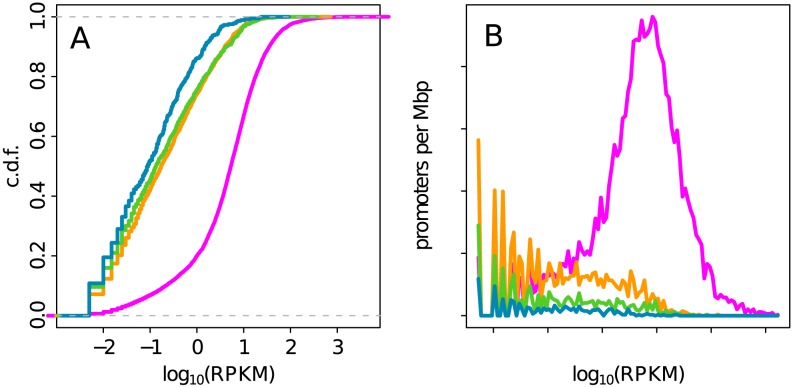
Gene expression in the four chromatin states. (A) c.d.f. of gene expression (measured in 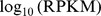, see Materials and Methods) in the four chromatin states. (B) Density of promoters in the 4 chromatin states as a function of gene expression (genes were grouped into bins of width 0.05 in 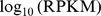 unit). Same color coding as in Fig. 3A.

To investigate gene expression in chromatin states, we used a data set of 17872 genes with a valid expression value in K562 (Materials and Methods). Of those genes, 15869 belong to one of the chromatin states. We found that a vast majority of expressed genes with a 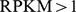 (Equation 7) are in the early replicating euchromatin state C1 (Fig. 7B), which confirms the link between MRT and expressed gene density previously reported in mammals [55], [58], [59], [61]. As expected, most of the genes in the facultative Pc repressed heterochromatin state C2 are non expressed. Interestingly, we found that the density of non expressed genes in C1 is equivalent to the one in C2, indicating that it is more the predominance of active genes that characterizes early replicating regions than the absence of repressed genes. This explains why the correlation between MRT and gene expression is stronger if one considers the expressed gene density (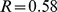, 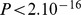)) than the mean expresssion (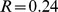, 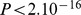) as previously observed in *Drosophila* [54]. Indeed in C1 the mean gene expression level is lowered by the presence of a non negligible set of non-expressed genes. The few genes in the heterochromatin states C3 and C4 are silent except a minority of them.

We assessed gene function on the basis of gene ontology [123]. We analyzed the genes in each chromatin states according to their biological process (Supplementary Fig. S3), component (Supplementary Fig. S4) and function (Supplementary Fig. S5) using GO SLIM annotation (Materials and Methods). We computed the enrichment p-value using the Hypergeometric distribution and used the odd ratio value to determine if the deviation from expected number of genes for the considered GO terms was an enrichment (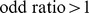) or a depletion (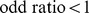). As previously observed for gene expression, these GO terms provide some clear discrimination between genes in the early replicating transcriptionally active euchromatin C1 and genes in the repressed heterochromatin states C2, C3 and C4. Genes enriched in C1 are almost systematically depleted in C2, C3 and C4, whereas on the opposite, genes that are depleted in C1 are enriched in at least one if not all the heterochromatin states C2, C3 and C4. We found C1 to be enriched mainly in housekeeping genes. The highest enrichments were obtained for the following process categories: mRNA processing, translation, ribosome biogenesis, DNA metabolic process, chromosome organization and segregation, cell cycle and cell division and for the corresponding component categories: ribosome, chromosome, nucleolus, nucleoplasm, nuclear envelope, mitochondrion and microtubule organizing center. The highly depleted process categories in C1 correspond to tissue specific genes that are not expressed in the immature myeloid K562 cell line as for example neurological system process, extracellular matrix organization, cell adhesion and cell motility, or that are defficient in these cancer cells like circulating system process [124], [125].

### Compositional content of chromatin states

**Figure 8:**
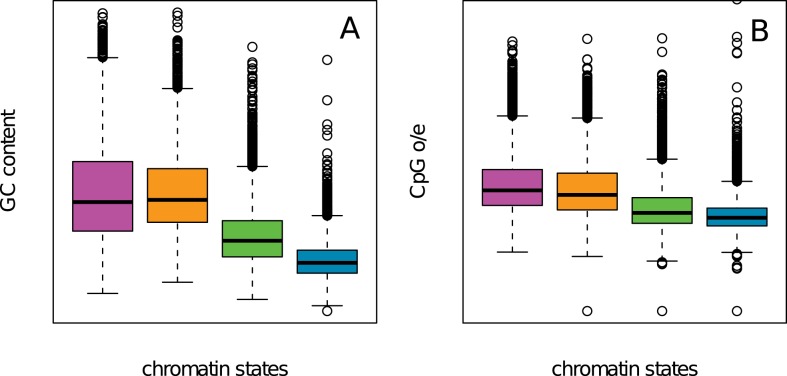
Sequence composition in the four chromatin states. (A) Boxplots of GC content computed in 100 kb non-overlapping windows per chromatin state. (B) Boxplots of CpG o/e computed in 100 kb non-overlapping windows per chromatin states. Same color coding as in Fig. 3A.

Along the line of the isochore model [126], GC-rich and GC-poor regions were shown to match the cytogenic R and G bands and to correlate well with early and late replicating domains in mammals [8], [127], [128]. GC-rich regions correspond to regions of very high density of genes including the housekeeping genes and associated CpG islands. This also correspond to regions enriched in short inter-dispersed repetitive DNA elements (SINEs, Alu) [8]. In contrast, GC-poor regions are definitely poor in genes, predominantly tissue-specific genes containing rather large introns, but are relatively rich in long inter-disperse repetitive DNA elements (LINES) [8] that are significantly more abundant in these regions. Consistently, we found that the early replicating euchromatin state C1 has a GC content distribution shifted to higher values as compared to the unmarked and constitutive heterochromatin states C3 and C4 respectively (Fig. 8A). C1 is definitely GC-rich with an mean value 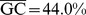 that is significantly higher than the genome average (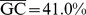). On the opposite C3 and C4 are GC-poor with 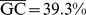 and 36.7%, respectively. Surprisingly, the Pc repressed facultative heterochromatin state C2 has a GC content distribution similar to the one obtained for C1 (Fig. 8) with 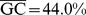. This means that if a high density of early replicating and highly expressed genes implies a high GC content, the reciprocal is not true. For example, C2 loci corresponding to 18% of the genome are GC-rich (Fig. 8A) but gene poor (Table 1) and most of these C2 genes are silenced by Pc proteins.

Cytosine DNA methylation is a mediator of gene silencing in repressed heterochromatin regions, while in potentially active open chromatin regions DNA is essentially unmethylated [129], [130]. Methyl-cytosines being hypermutable, prone to deamination to thymines, CpG o/e ratio (Materials and Methods) is commonly used as an estimator of DNA methylation, the higher this ratio, the lower the methylation [101], [131]. When computing CpG o/e after removing the CpG islands (CGIs) that are short unmethylated regions rich in CpG, in the four chromatin states, we found a significant shift of the CpG o/e pdf to smaller values when going from C1 (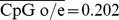) to C2 (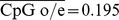), C3 (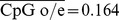) and C4 (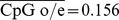) (Fig. 8). Thus relative to the genome average value 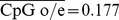, the early replicating transcriptionally active euchromatin state C1 is clearly hypomethylated. The mid-S repressed facultative heterochromatin state C2 is also, but at a lesser extent, less methylated than the entire genome. As expected the late replicating unmarked and constitutive heterochromatin states C3 and C4 are definitely methylated, the later being significantly more methylated than the entire genome. Thus the differences in CpG o/e (Fig. 8B) and MRT (Fig. 6A) observed in the four chromatin states C1, C2, C3 and C4, explain the significant correlation observed genome wide between methylation and replication timing (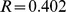, 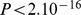)) [101].

Note that chromatin state compositional content in Monocd14ro1746 is quite the same as in K562 (Supplementary Fig. S11). In constrast, C3 and C4 in GM12878 have exchanged their GC and CpGo/e distributions (Supplementary Fig. S9). Interestingly, this phenomenon is paired with C3 becoming more late in GM12878 than C4 (Supplementary Fig. S9). This observation suggests that the genomic regions that replicate late in S phase are more likely specified by sequence features than by epigenetic features. However, the GC content cannot be the primary determinant of MRT for C1 and C2 states. Indeed the GC distributions in C1 and C2 are nearly the same (Fig. 8A, Supplementary Fig. S9A and S11A) whereas a great discrepancy is observed in the MRT distributions (Fig. 6, Supplementary Fig. S8 and MRT data non available).

### Repartition of chromatin states along human chromosomes

**Figure 9:**
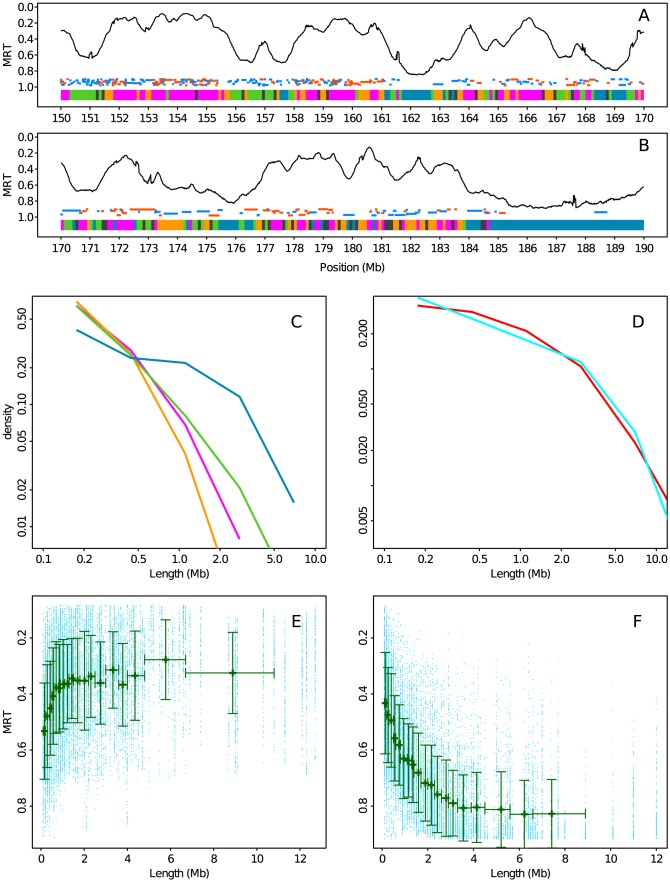
Genome-wide spatial distribution of the four chromatin states. (A) MRT profile along a 20 Mb long fragment of human chromosome 1. Below the MRT profile, gene positions are indicated by a segment (blue: not expressed, orange: expressed). At the bottom of the plot, the chromatin state of each 100 kb window is represented using the same color coding as in Fig. 3A. (B) Same as (A) for the following 20 Mb fragment of the human chromosome 1. (C) Histogram of chromatin state block length in a logarithmic representation (see Materials and Methods). (D) Same as (C) for chromatin state blocks formed by states 1 and 2 (1+2, light red) or by states 3 and 4 (3+4, light blue). (E) MRT in chromatin state blocks (1+2) with respect to their length. Each 100 kb window in a chromatin state block is represented by a blue dot. The mean profile was obtained by (i) ordering data points according to their block length, (ii) grouping them in classes of equal number of data points and (iii) computing the average length and MRT over each class. Vertical bars represent the standard deviation. Horizontal bars represent the range of length over each class. (F) Same as (E) for chromatin state blocks (3+4).

Once mapped on the genome (Fig. 9A,B), the four prevalent chromatin states differ not so much in the genome coverage but mainly in their number and length distribution of domains or blocks of adjacent 100-kb-loci in the same chromatin state (Table 2 and Fig. 9C). C1 and C2 chromatin blocks are more numerous but they are shorter with a mean length 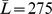 kb and 228 kb respectively. Their length pdfs do not reveal many domains larger than 1 Mb. C3 chromatin blocks are slightly less numerous and also mostly short, the larger mean length 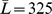 kb resulting from the existence of a few large C3 streches of several Mb length. The C4 block length pdf definitely differs from the previous ones by the presence of a fat tail. Not only the mean length 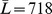 kb is about three times the ones of C1, C2 blocks, but most of the C4 domains exceed 1 Mb up to 5 Mb and more, hence they are less numerous (Fig. 9C). This observation is quite consistent with the HP1-associated classical heterochromatin spreading mechanism and its possible association with the nuclear envelope [3], [6].

When looking at the distribution of chromatin states along human chromosomes (Fig. 9A,B), there is a clear evidence that C1, C2, C3 and C4 blocks are not distributed independently. In large regions with MRT ≲ 
0.4, short C1 and C2 blocks intersperse with each other, the C1s being the earliest ones (*e.g* from 158 to 161 Mb in Fig. 9A). In a few 100 kb wide regions of MRT
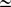
0.6, C3 blocks are observed with a repressive effect (*e.g* around 156 Mb in Fig. 9A where chromosome 1 contains a lot of olfactory receptor genes). C4 lies in very late regions MRT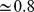 and form large uninterrupted blocks of several Mb size (*e.g* from 185 to 190 Mb in Fig. 9A). This MRT dependent spatial organization of chromatin states prompted us to investigate neighborhood dependency between 100 kb loci. The obtained transition matrix (Table 3) confirms that C4 loci have by far the highest probability (0.85) to have a C4 neighbor consistent with C4 blocks being much longer than the other chromatin state blocks (Table 2 and Fig. 9C). It also quantifies the fact that C1 loci (and in turn blocks) have a much higher probability to have a neighbor that is a C2 locus (block) than a C3 or C4 locus (block) and vice-versa. This is consistent with the fact that C1 and C2 are likely to be replicated one after each other in early and mid S phase whereas C3 and C4 are replicated much later (Fig. 6). Consistently C4 loci (blocks) have a highest probability to have a neighbor that is a C3 locus (block) whereas C3 loci (blocks) have apparently no special preference. The spatial organization of chromatin blocks suggests that we can associate C1+C2 on one side and C3+C4 on the other side (Supplementary Fig. S2B) resulting in large-scale blocks of surprisingly very similar length distributions (Fig. 9D) with fat tails and respective means 779 kb and 808 kb. These mega-base long C1+C2 and C3+C4 chromatin blocks would on average be replicated rather early (Fig. 9E) and late (Fig. 9F), respectively. Importantly, fixing the number of chromatin states to two in our PCA and cluster analysis does not result in the same dichotomic picture (Supplementary Fig. S2A). Instead we discriminate the active chromatin state C1 from a composite silent state C2+C3+C4 (Supplementary Fig. S2B)

**Table 3:** Transition matrix between chromatin states.

	C1	C2	C3	C4	D
	0.22	0.18	0.22	0.22	0.16
from C1	0.59	0.21	0.082	0.024	0.094
from C2	0.27	0.51	0.097	0.017	0.11
from C3	0.084	0.078	0.65	0.079	0.11
from C4	0.024	0.013	0.077	0.85	0.035
from D	0.13	0.12	0.15	0.05	0.55

The first line is the probability of each chromatin state. The matrix below the first line is the Markov transition matrix between states (see Materials and Methods for its estimation). A value at the 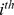 row and the 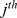 column is the probability to find the chromatin state j in a 100 kb window next to a 100 kb window of chromatin state i. D corresponds to 100 kb windows that are not classified in any chromatin state.

Note that when using the so-computed transition matrix between chromatin states (Table 3) to generate randomly synthetic chromosomes, we obtained very good predictions for the four chromatin state block mean lengths (Table 2). However the corresponding sample standard deviations so predicted are significantly smaller than the ones computed for the genuine human chromosomes which is an indication that the succession of chromatin states along human chromosomes is probably governed by a more global and elaborated underlying segmentation process.

### Distribution of chromatin states inside replication timing U-domains

**Figure 10:**
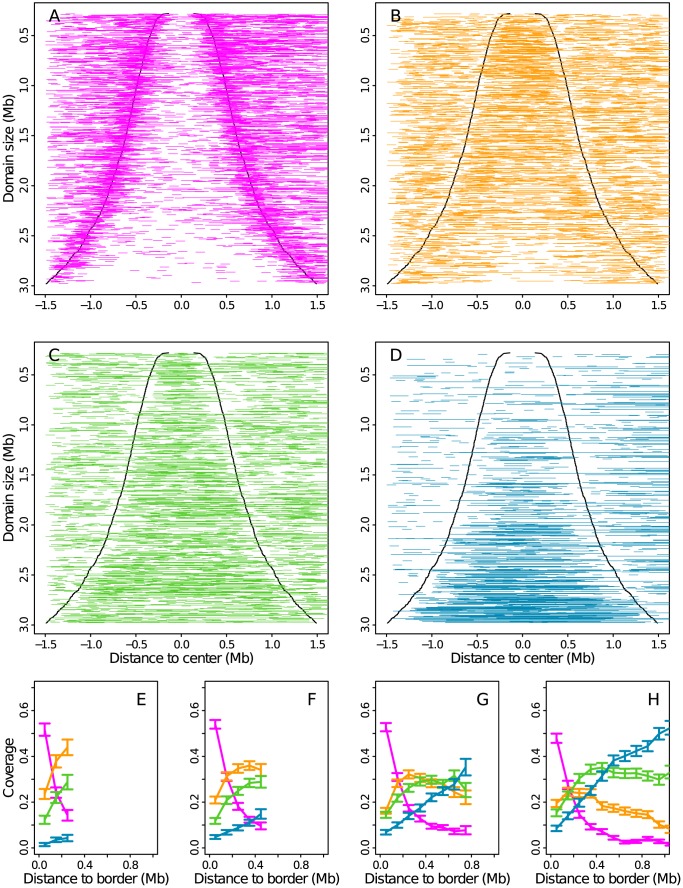
Distribution of the four chromatin states inside replication timing U-domains. (A) The 876 K562 U-domains were centered and ordered vertically from the smallest (top) to the largest (bottom). All transcriptionally active chromatin state C1 100-kb-windows were represented by an horizontal segment of the corresponding length. (B) Same as (A) for the Pc repressed by chromatin state C2. (C) Same as (A) for the silent unmarked chromatin state C3. (D) Same as (A) for the HP1 heterochromatin state C4. (E) Mean coverage of chromatin state with respect to the distance to the closest U-domain border for U-domains smaller than 0.8 Mb. Error bars represent the standard deviation of the mean. (F) Same as (E) for U-domains of size between 0.8 Mb and 1.2 Mb. (G) Same as (E) for U-domains of size between 1.2 Mb and 1.8 Mb. (H) Same as (E) for U-domains of size between 1.8 Mb and 3.0 Mb. Same color coding as in Fig. 3A.

When concentrating our study on the 876 replication timing U-domains previously identified in K562 cells [80], we revealed some remarkable organization of the four prevalent chromatin states (Fig. 10A). The highly expressed gene rich euchromatin state C1 is found to be confined in a closed (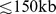) neighborhood of the “master” replication origins that border each individual U-domains (Fig. 10A). As confirmed on the mean occupation profiles obtained for four U-domains size categories (Fig. 10 E, F, G, H), this confinement is independent of the U-domains size and consistent with the previous observation [80], [101] that U/N-domain borders are significantly enriched in DNase I hypersensitive sites and in insulator-binding proteins CTCF. C1 can thus be seen as specifying the early initiation zones that border U-domains and that were further shown [80] to delimit topological domains on genome-wide (Hi-C) chromatin state conformation data. The Pc repressed heterochromatin state C2 is mostly found at finite distance (∼200–300 kb) from U-domain borders as clearly seen on the largest U-domains whose centers are drastically devoided of C2 loci (Fig. 10B,H). In small U-domains (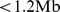), C2 occupies in majority their centers (Fig. 10E,F) that are replicated in mid-S phase. U-domain borders are also significantly depleted in unmarked and constitutive heterochromatin states C3 (Fig. 10C) and C4 (Fig. 10D), respectively. C3 is already present in the center of small U-domains (Fig. 10E,F) and homogeneously occupies large U-domain centers (Fig. 10G,H). C4 is significantly found in the center of U-domains that are larger than 1 Mb; C4 spreads and becomes predominant when increasing the size of U-domains beyond 1.8 Mb (Fig. 10G,H). These results show that the replication “wave” starting from the early initiation zones at U-domain borders and propagating inside U-domains during S-phase with the progressive activation of secondary replication origins [79], actually corresponds to a directional path through the four prevalent chromatin states C1, C2, C3 and ultimately C4 in the largest U-domains. This gradient of chromatin structure, from active openess at U-domain borders to closeness at U-domain centers via intermediate Pc repressed and unmarked heterochromatins is likely to be a key ingredient in the long-range chromatin control of the spatio-temporal replication program that underlies the megabase-sized replication fork polarity gradients observed in about 50% of the human genome [79], [80].

### Conclusion/perspectives

In summary, this integrative analysis of epigenetic mark maps in the immature myeloid human cell line K562 has shown that the combinatorial complexity of these epigenetic data can be reduced to four prevalent chromatin states, one transcriptionally active open euchromatin state C1 and three distinct and silent heterochromatin states, namely a Pc repressed state C2, a unmarked silent state C3 and a HP1-associated constitutive state C4. By performing this statistical study at the (low) resolution 100 kb of available genome-wide MRT data, we have found that these chromatin states actually replicate at distinct periods of the S-phase, C1 replicates early, C2 is a mid-S phase phase state whereas C3 replicates later than C2 but before C4 that replicates very late, almost at the end of S-phase. In the Supplementary Data are reported, for comparison, the results of a similar integrative analysis of epigenomic data in the lymphoblastoid cell line GM12878 (Supplementary Figs S6, S7, S8 and S9) and in the blood cell line Monocd14ro1746 (Supplementary Figs S10, S11), which confirm that the classification of the human epigenome in four main chromatin states likely summarizes the data in different cell types. Interestingly, these four main chromatin states display remarkable similarities with that found in different cell types in *Drosophila* [21] and *Arabidopsis* [19] at the resolution ∼1 kb of gene expression data, suggesting the existence of simple principles of organization in metazoans as well as in plants [19]–[22]. When mapping these four chromatin states along the human chromosomes, our study reveals that the human genome can be segmented into megabase-sized domains of three different types with distinct spatio-temporal replication programs. In 50% of the human genome that are covered by the replication U-domains [80], the U-shape of the replication timing profile indicates that the effective replication velocity (which equals the inverse of the replication timing derivative [80], [98]) increases from U-domain borders to centers [79] as the signature of an increasing origin firing frequency during S-phase [132]. Our results (Fig. 10) show that this acceleration of the replication wave is actually observed along a directional path through the four main chromatin states, the open euchromatin state C1 at U-domain borders successively followed by the heterochromatin states C2, C3 and C4 at the U-domain centers. To which extent this chromatin gradient influences fork progression from the “master” early initiation zones at U-domain borders and secondary origins activation inside U-domains is a key issue of current modeling [79], [133]–[135] of the spatio-temporal replication program in human and more generally in mammals. The complete analysis of the other half of the human genome that is complementary to U-domains is more in agreement with the traditional dichotomic picture proposed in early studies of the mouse [55]–[57] and human [59], [65], [75] genomes, where early and late replicating regions occur in separated compartments of open and close chromatin, respectively. About 25% of the human genome are covered by megabase sized GC-rich (C1+C2) chromatin blocks that on average replicate early by multiple almost synchronous origins with equal proportion of forks coming from both directions (Table 4). This absence of well-positioned origins explains that the skew has not accumulated in these gene-rich regions that were shown to be devoided of skew N-domains [90]–[93]. The last 25% of the human genome corresponds to megabase sized GC-poor domains of interspersed (C3+C4) heterochromatin states or of long C4 domains that on average replicate late by again multiple almost coordinated origins (Table 4). These gene-poor regions are also devoided of skew N-domains and can be seen as the late replicating counter-part of the gene-rich (C1+C2) regions.

**Table 4:** Distribution of chromatin states outside replication timing U-domains.

Chromatin states	C1	C2	C3	C4	C1+C2	C3+C4
total length(Mb)	446.3	221.8	295.2	388.8	750.6	745.5
Number	1955	1350	1216	542	1336	1031
mean(length)	228.3	164.3	242.7	717.4	561.8	723.1
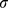 (length)	218.2	133.6	435.0	1035.4	602.0	1275.1
M0 mean	134	115	121	130		

Same as the five first lines of Table 2 after removing the replication U-domains from the analysis.

Extending this study to different cell types including ES, somatic and cancer cells looks very promising. By performing our integrative analysis at low (100 kb) and high (1 kb) resolutions in parallel, we should be in position to investigate the global reorganization of replication domains during differentiation (or disease) in relation to coordinated changes in chromatin state and gene expression. For example, this multivariate approach should shed a new light on the so-called replication domain “consolidation” phenomenon [56] that corresponds to the disappearance (EtoL transition) or appearance (LtoE transition) of a U-domain border during differentiation [80].The probable coordinated change in chromatin state at 100 kb resolution and the possible change at 1 kb resolution are likely to explain the possible change in gene expression. This opens new perspectives in the study of chromatin-mediated epigenetic regulation of transcription and replication in mammalian genomes in both health and disease.

## Materials and Methods

### Mean replication timing data and replication U-domain coordinates

Timing profiles for the immature myeloid cell line K562 and the lymphoblastoid cell line GM06990 were obtained from the authors [80]. The mean replication timing (MRT) is given for 27656 100 kb non-overlapping windows in hg18 coordinates. We also retrieved the coordinates of the 876 U-domains in K562 and 882 U-domains in GM06990 from the authors [80].

### Histone marks, H2AZ, CTCF, RNAP II, Sin3A and CBX3 ChIP-seq data

For all ChIP-seq data, we downloaded data in the Encode standard format “broadpeaks” (http://genome.ucsc.edu/FAQ/FAQformat.html). Broadpeaks format is a table of significantly enriched genomic intervals. Most of the data correspond to the release 3 (August 2012) of the Broad histone track. We downloaded the tables from: http://hgdownload.cse.ucsc.edu/goldenPath/hg19/encodeDCC/wgEncodeBroadHistone/. The CBX3 and Sin3A data corresponds to the release 3 (September 2012) of the HAIB TFBS track. Tables were downloaded from the UCSC from: http://hgdownload.cse.ucsc.edu/goldenPath/hg19/encodeDCC/wgEncodeHaibTfbs/

For the K562 cell line, we downloaded the broadpeak tables for the following antibodies: CTCF, H3K27ac, H3K27me3, H3K36me3, H3K4me3, H3K9me3, RNAP ll, H2AZ, H3K79me2, H3K9me1, H4K20me1, CBX3, Sin3A. For the GM12878 cell line, we downloaded: CTCF, H3K27ac, H3K27me3, H3K36me3, H3K4me3, H3K9me3. For the Monocd14ro1746 cell line we downloaded: CTCF, H2AZ, H3K27ac, H3K27me3, H3K36me3, H3K4me3, H3K79me2, H3K9ac, H3K9me3. Genomic intervals were then mapped back to hg18 using LiftOver.

### Epigenetic profile computation at 100 kb resolution

For each ChIP-seq data, we computed a profile at the 100 kb resolution for the 27656 non-overlapping windows for which MRT is defined. The read density for one antibody in a window is the number of reads in this window that fall in significantly enriched intervals normalized by the window length.

### Rank transformation and Spearman correlation matrix

All statistical computations were performed using the R software (http://www.r-project.org/).

In order to compute the Spearman correlation matrix, the epigenetic profiles at 100 kb resolution were transformed with the R function *rank* with option *ties.method = max*. Then we computed the Pearson correlation matrix on the transformed dataset. To reorder the matrix in Fig. 1, we computed the Spearman correlation distance 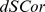 as:
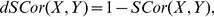
(1)where 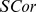 is the spearman correlation. Then, a dendrogram was computed using the R function *hclust* with option *method = average* and with 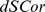 as dissimilarity.

### Principal component analysis

Principal component analysis was performed on the rank transformed dataset using the function *dudi.pca* from the R package *ade4* (see http://pbil.univ-lyon1.fr/ADE-4 and Ref. [107]) with the option *scale = TRUE* (*i.e.* each variable is centered and normalized before the PCA computation). The first three components were retained which accounts for 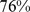 of the dataset variance (see Supplementary Fig. S1), and clustering was performed in this 3D space.

### Clustering strategy

We used Clara algorithm [88] which is an optimization of k-means for large data set. We used the *clara* function implemented in the R package *cluster*. The options were set to: *stand = FALSE, sampsize = 500, samples = 20, metric = euclidean*.

To assess the number of clusters, we used the pooled within-cluster sum of squares around the cluster mean. Suppose that the data set of size 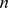 is divided in k clusters 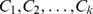. Let d(x,y) be the euclidean distance between the points x and y. Let 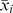 be the mean of the 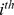 cluster, then the within-cluster sum of squares for this cluster is:
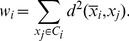
(2)


The pooled within-sum of squares for the k clusters is:
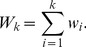
(3)


The pooled within-cluster sum of squares necessarily decreases with the number of clusters. A good choice for the number of clusters is the critical point where some clear crossover is observed from a fast decrease of 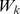 at small k values to a weak decrease of 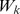 at large k values. This means that, after this critical point, no much information is gained by adding a new cluster. In our analysis this crossover occurs for k = 4 clusters (see Fig. 3B).

We also used the Gap statistic [109] which is defined by :
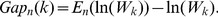
(4)



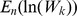
 is the expected value of 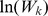 for a sample of size 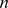 drawn from a proper reference distribution. We choose, as a reference, a uniform distribution over the range of the observed data. A good choice for the number of clusters is a value of k so that 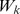 is much smaller than the expected 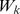 from a random distribution (*i.e.* a high value of 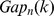). Four clusters is also a reasonable choice according to the gap statistic index computed with R package *clusterSim* (see Fig. 3C).

Poorly clustered data points were removed from the set of chromatin states. The silhouette value [108] is a way to quantify how well a point is clustered.

**Definition 1.** *Given a particular clustering,* 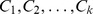 *, of the data in k clusters, let i be a data point and* 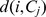 *the average distance of the data point i to the members of the cluster* 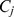 *. Let i be a member of cluster* 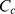 *and*

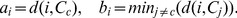
(5)
*The silhouette value of the data point i is defined as:*

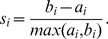
(6)


A silhouette value below 0 means that the data point is actually closer in average to the points from another cluster than to the one it has been assigned to. Points with a negative silhouette value are border line allocations. We decided to remove those points from the set of identified chromatin states. Hence chromatin states are groups (clusters) with homogeneous epigenetic features. 91% of all 100 kb non-overlapping windows of the human genome were assigned to one of the four chromatin states C1, C2, C3 or C4.

### Markov transition matrix estimation

The number of transitions from i to j, 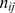, is the number of 100 kb windows of state i contiguous to a window of state j (the sense or antisense orientation is not taken in account). Let 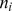 be the number of windows in chromatin state i. The conditional probability of a transition from i to j given i is 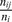.

### Annotation and expression data

As human gene coordinates, we used the UCSC Known Genes table. When several genes presenting the same orientation overlapped, they were merged into one gene whose coordinates corresponded to the union of all the overlapping gene coordinates, resulting in 23818 distinct genes.

Expression data were retrieved from the Genome Browser of the University of California Santa Cruz (UCSC). To construct our expression data set, we used RefSeq Genes track as human gene coordinates. Genes with alternative splicing were merged into one transcript by taking the union of exons. Hence the TSS was placed at the beginning of the first exon. We obtained a table of 23329 genes. We downloaded expression valuess from the release 2 of Caltech RNA-seq track (ENCODE project at UCSC: http://hgdownload.cse.ucsc.edu/goldenPath/hg18/encodeDCC/wgEncodeCaltechRnaSeq/).

Expression for one transcript is given in reads per kilobase of exon model per million mapped reads (RPKM) [136]. RPKM is defined as:
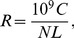
(7)where C is the number of mappable reads that fall into gene exons (union of exons for genes with alternative splicing), N is the total number of mappable reads in the experiment, and L is the total length of the exons in base pairs. We associated 17872 genes with a valid RPKM value in K562.

### CpG o/e computation and GC content

CpG observed/expected ratio (CpG o/e) was computed as 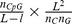, where 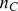, 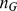 and 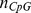 are the numbers of C, G and dinucleotides CG, respectively, counted along the sequence, L is the number of nonmasked nucleotides and *l* is the number of masked nucleotide gaps plus one, *i.e.* *L*-*l* is the number of dinucleotide sites. The CpG o/e was computed over the sequence after masking annotated CGIs. The GC content was computed on the native sequence.

### Chromatin state blocks

We detected contiguous windows of the same chromatin state (C1 to C4). We then kept the coordinates of the blocks of contiguous windows. To form chromatin state blocks of states (1+2), we merely detected contiguous windows of state 1 or 2. The same procedure was applied to define chromatin blocks of states (3+4). For chromatin blocks (1+2) and (3+4), we authorized the inclusion of isolated windows which don't belong to any chromatin state so to not disrupt very long blocks.

### GO term enrichment

Each gene name of our annotation dataset was associated to several GO terms from GO SLIM (high level GO terms) using the online mapper: http://go.princeton.edu/cgi-bin/GOTermMapper. Then for each chromatin state (C1 to C4), the number of occurrences of each GO term was determined by the number of promoters belonging to that state and associated to this GO term. The enrichment for each GO term in each cluster was tested using Fisher's exact test. We applied a procedure to control the false discovery rate (FDR) as described in [137]. The upper limit of the FDR was fixed to 20%. After detecting significant deviation from a random repartition of GO term occurrences, we used the odd ratio value to determine if the deviation was an enrichment (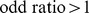) or a depletion (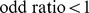).

## Supporting Information

Figure S1 **PCA analysis.** (A) Percentage of variance accounted by the first thirteen principal components ordered according to their corresponding variance (eigenvalues). (B) Cumulative variance.
(EPS)

Figure S2 **Dichotomic analysis with two chromatin states.** (A) Results of our clustering procedure when using two clusters (the number of clusters is the only parameter of the procedure). We found a segmentation between transcriptionally active chromatin (red) and silent chromatin (blue). (B) Same representation for chromatin state blocks (1+2) (light red) and (3+4) (light blue) as defined in Fig. 9.
(EPS)

Figure S3
**GO term enrichment of the Biological Process ontology in the four chromatin states.** Fisher's exact test odd ratios were computed for each GO term of the Biological Process ontology in the four chromatin states. If the test was unsignificant the corresponding cell was left blank (see Materials and Methods) otherwise the 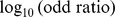 value was coded using the color map shown at the bottom.(EPS)

Figure S4 **GO term enrichment of the Cellular Component ontology in the four chromatin states.** Same as Fig. S3 for the Cellular Component GO term annotation.
(EPS)

Figure S5 **GO term enrichment of the Molecular Function ontology in the four chromatin states.** Same as Fig. S3 for the Molecular Function GO term annotation.
(EPS)

Figure S6 **Defining the four chromatin states for the GM12878 cell line.** Scatterplot of the data points onto the first three principal components for the GM12878 cell line. Color dots indicate the four chromatin states as found by our clustering procedure (pink: transcriptionally active chromatin, orange: chromatin repressed by polycomb, green: silent unmarked chromatin, blue: HP1 heterochromatin). Points in dark grey are not classified in any chromatin state (see Materials and Methods).
(EPS)

Figure S7 **Repartition of epigenetic marks in the four chromatin states for the GM12878 cell line.** Boxplots of the decimal logarithm of epigenetic mark ChIP-seq read density in 100 kb non-overlapping windows per chromatin state. Same color coding as in Fig. S6.
(EPS)

Figure S8 **MRT in the four chromatin states for the GM12878 cell line.** (A) Boxplots of MRT computed in 100 kb non-overlapping windows per chromatin state. (B) Empirical cumulative distribution function (c.d.f.) of MRT in the four chromatin states. Same color coding as in Fig. S6.
(EPS)

Figure S9 **Sequence composition in the four chromatin states in the GM12878 cell line.** (A) Boxplots of GC percent computed in 100 kb non-overlapping windows per chromatin state. (B) Boxplots of CpG o/e computed in 100 kb non-overlapping windows per chromatin states. Same color coding as in Fig. S6.
(EPS)

Figure S10 **Repartition of epigenetic marks in the four chromatin states for the Monocd14ro1746 cell line.** Boxplots of the decimal logarithm of epigenetic mark ChIP-seq read density in 100 kb non-overlapping windows per chromatin state. Same color coding as in Fig. S6.
(EPS)

Figure S11 **Sequence composition in the four chromatin states in the Monocd14ro1746 cell line.** (A) Boxplots of GC percent computed in 100 kb non-overlapping windows per chromatin state. (B) Boxplots of CpG o/e computed in 100 kb non-overlapping windows per chromatin states. Same color coding as in Fig. S6
(EPS)

## References

[1] Chakalova L, Debrand E, Mitchell JA, Osborne CS, Fraser P (2005) Replication and transcription: shaping the landscape of the genome. Nat Rev Genet 6: 669–677. 16094312 10.1038/nrg1673

[2] Kouzarides T (2007) Chromatin modifications and their function. Cell 128: 693–705. 17320507 10.1016/j.cell.2007.02.005

[3] Maric C, Prioleau MN (2010) Interplay between DNA replication and gene expression: a harmonious coexistence. Curr Opin Cell Biol 22: 277–283. 20363609 10.1016/j.ceb.2010.03.007

[4] Gilbert DM (2010) Evaluating genome-scale approaches to eukaryotic DNA replication. Nat Rev Genet 11: 673–684. 20811343 10.1038/nrg2830 PMC2962615

[5] Arneodo A, Vaillant C, Audit B, Argoul F, d'Aubenton-Carafa Y, et al (2011) Multi-scale coding of genomic information: From DNA sequence to genome structure and function. Phys Rep 498: 45–188.

[6] Zhou VW, Goren A, Bernstein BE (2011) Charting histone modifications and the functional organization of mammalian genomes. Nat Rev Genet 12: 7–18. 21116306 10.1038/nrg2905

[7] Bickmore WA, van Steensel B (2013) Genome architecture: domain organization of interphase chromosomes. Cell 152: 1270–1284. 23498936 10.1016/j.cell.2013.02.001

[8] Lander ES (2001) Initial sequencing and analysis of the human genome. Nature 409: 860–921. 11237011 10.1038/35057062

[9] Schones DE, Zhao K (2008) Genome-wide approaches to studying chromatin modifications. Nat Rev Genet 9: 179–191. 18250624 10.1038/nrg2270 PMC10882563

[10] Rando OJ, Chang HY (2009) Genome-wide views of chromatin structure. Annu Rev Biochem 78: 245–271. 19317649 10.1146/annurev.biochem.78.071107.134639 PMC2811691

[11] Roudier F, Teixeira FK, Colot V (2009) Chromatin indexing in Arabidopsis: an epigenomic tale of tails and more. Trends Genet 25: 511–517. 19850370 10.1016/j.tig.2009.09.013

[12] Feng S, Jacobsen SE (2011) Epigenetic modifications in plants: an evolutionary perspective. Curr Opin Plant Biol 14: 179–186. 21233005 10.1016/j.pbi.2010.12.002 PMC3097131

[13] Gerstein MB, Lu ZJ, Van Nostrand EL, Cheng C, Arshinoff BI, et al (2010) Integrative analysis of the *Caenorhabditis elegans* genome by the modENCODE project. Science 330: 1775–1787. 21177976 10.1126/science.1196914 PMC3142569

[14] The modENCODE Consortium (2010) Identification of functional elements and regulatory circuits by Drosophila modENCODE. Science 330: 1787–1797. 21177974 10.1126/science.1198374 PMC3192495

[15] Kharchenko PV, Alekseyenko AA, Schwartz YB, Minoda A, Riddle NC, et al (2010) Comprehensive analysis of the chromatin landscape in *Drosophila melanogaster* . Nature 471: 480–485. 21179089 10.1038/nature09725 PMC3109908

[16] Bernstein BE, Meissner A, Lander ES (2007) The mammalian epigenome. Cell 128: 669–681. 17320505 10.1016/j.cell.2007.01.033

[17] The ENCODE Project Consortium (2011) A user's guide to the encyclopedia of DNA elements (encode). PLoS Biol 9: e1001046. 21526222 10.1371/journal.pbio.1001046 PMC3079585

[18] The ENCODE Project Consortium (2007) Identification and analysis of functional elements in 1% of the human genome by the ENCODE pilot project. Nature 447: 799–816. 17571346 10.1038/nature05874 PMC2212820

[19] Roudier F, Ahmed I, Bérard C, Sarazin A, Mary-Huard T, et al (2011) Integrative epigenomic mapping defines four main chromatin states in Arabidopsis. EMBO J 30: 1928–1938. 21487388 10.1038/emboj.2011.103 PMC3098477

[20] Liu T, Rechtsteiner A, Egelhofer TA, Vielle A, Latorre I, et al (2011) Broad chromosomal domains of histone modification patterns in *C.elegans* . Genome Res 21: 227–236. 21177964 10.1101/gr.115519.110 PMC3032926

[21] Sexton T, Yaffe E, Kenigsberg E, Bantignies F, Leblanc B, et al (2012) Three-dimensional folding and functional organization principles of the *Drosophila* genome. Cell 148: 458–472. 22265598 10.1016/j.cell.2012.01.010

[22] Filion GJ, van Bemmel JG, Braunschweig U, Talhout W, Kind J, et al (2010) Systematic protein location mapping reveals five principal chromatin types in *Drosophila* cells. Cell 143: 212–224. 20888037 10.1016/j.cell.2010.09.009 PMC3119929

[23] Ernst J, Kellis M (2010) Discovery and characterization of chromatin states for systematic annotation of the human genome. Nat Biotechnol 28: 817–825. 20657582 10.1038/nbt.1662 PMC2919626

[24] Hon G, Wang W, Ren B (2009) Discovery and annotation of functional chromatin signatures in the human genome. PLoS Comput Biol 5: e1000566. 19918365 10.1371/journal.pcbi.1000566 PMC2775352

[25] Wang Z, Schones DE, Zhao K (2009) Characterization of human epigenomes. Curr Opin Genet Dev 19: 127–134. 19299119 10.1016/j.gde.2009.02.001 PMC2699568

[26] Lee BK, Bhinge AA, Battenhouse A, McDaniell RM, Liu Z, et al (2012) Cell-type specific and combinatorial usage of diverse transcription factors revealed by genome-wide binding studies in multiple human cells. Genome Res 22: 9–24. 22090374 10.1101/gr.127597.111 PMC3246210

[27] Ernst J, Kheradpour P, Mikkelsen TS, Shoresh N, Ward LD, et al (2011) Mapping and analysis of chromatin state dynamics in nine human cell types. Nature 473: 43–49. 21441907 10.1038/nature09906 PMC3088773

[28] Ram O, Goren A, Amit I, Shoresh N, Yosef N, et al (2011) Combinatorial patterning of chromatin regulators uncovered by genome-wide location analysis in human cells. Cell 147: 1628–1639. 22196736 10.1016/j.cell.2011.09.057 PMC3312319

[29] Berezney R, Dubey DD, Huberman JA (2000) Heterogeneity of eukaryotic replicons, replicon clusters, and replication foci. Chromosoma 108: 471–484. 10794569 10.1007/s004120050399

[30] Bell SP, Dutta A (2002) DNA replication in eukaryotic cells. Annu Rev Biochem 71: 333–374. 12045100 10.1146/annurev.biochem.71.110601.135425

[31] Gilbert DM (2001) Making sense of eukaryotic DNA replication origins. Science 294: 96–100. 11588251 10.1126/science.1061724 PMC1255916

[32] Méchali M (2010) Eukaryotic DNA replication origins: many choices for appropriate answers. Nat Rev Mol Cell Biol 11: 728–738. 20861881 10.1038/nrm2976

[33] Bogan JA, Natale DA, Depamphilis ML (2000) Initiation of eukaryotic DNA replication: conservative or liberal? J Cell Physiol 184: 139–150. 10867638 10.1002/1097-4652(200008)184:2<139::AID-JCP1>3.0.CO;2-8

[34] Méchali M (2001) DNA replication origins: from sequence specificity to epigenetics. Nat Rev Genet 2: 640–645. 11483989 10.1038/35084598

[35] McNairn AJ, Gilbert DM (2003) Epigenomic replication: linking epigenetics to DNA replication. Bioessays 25: 647–656. 12815720 10.1002/bies.10305

[36] Aladjem MI (2007) Replication in context: dynamic regulation of DNA replication patterns in metazoans. Nat Rev Genet 8: 588–600. 17621316 10.1038/nrg2143

[37] Courbet S, Gay S, Arnoult N, Wronka G, Anglana M, et al (2008) Replication fork movement sets chromatin loop size and origin choice in mammalian cells. Nature 455: 557–560. 18716622 10.1038/nature07233

[38] Hamlin JL, Mesner LD, Lar O, Torres R, Chodaparambil SV, et al (2008) A revisionist replicon model for higher eukaryotic genomes. J Cell Biochem 105: 321–329. 18680119 10.1002/jcb.21828 PMC2574905

[39] Costas C, de la Paz Sanchez M, Stroud H, Yu Y, Oliveros JC, et al (2011) Genome-wide mapping of *Arabidopsis thaliana* origins of DNA replication and their associated epigenetic marks. Nat Struct Mol Biol 18: 395–400. 21297636 10.1038/nsmb.1988 PMC3079358

[40] Cayrou C, Coulombe P, Vigneron A, Stanojcic S, Ganier O, et al (2011) Genome-scale analysis of metazoan replication origins reveals their organization in specific but flexible sites defined by conserved features. Genome Res 21: 1438–1449. 21750104 10.1101/gr.121830.111 PMC3166829

[41] Sequeira-Mendes J, Diaz-Uriarte R, Apedaile A, Huntley D, Brockdorff N, et al (2009) Transcription initiation activity sets replication origin efficiency in mammalian cells. PLoS Genet 5: e1000446. 19360092 10.1371/journal.pgen.1000446 PMC2661365

[42] Lucas I, Palakodeti A, Jiang Y, Young DJ, Jiang N, et al (2007) High-throughput mapping of origins of replication in human cells. EMBO Rep 8: 770–777. 17668008 10.1038/sj.embor.7401026 PMC1978075

[43] Cadoret JC, Meisch F, Hassan-Zadeh V, Luyten I, Guillet C, et al (2008) Genome-wide studies highlight indirect links between human replication origins and gene regulation. Proc Natl Acad Sci USA 105: 15837–15842. 18838675 10.1073/pnas.0805208105 PMC2572913

[44] Karnani N, Taylor CM, Malhotra A, Dutta A (2010) Genomic study of replication initiation in human chromosomes reveals the inuence of transcription regulation and chromatin structure on origin selection. Mol Biol Cell 21: 393–404. 19955211 10.1091/mbc.E09-08-0707 PMC2814785

[45] Mesner LD, Valsakumar V, Karnani N, Dutta A, Hamlin JL, et al (2011) Bubble-chip analysis of human origin distributions demonstrates on a genomic scale significant clustering into zones and significant association with transcription. Genome Res 21: 377–389. 21173031 10.1101/gr.111328.110 PMC3044852

[46] Martin MM, Ryan M, Kim R, Zakas AL, Fu H, et al (2011) Genome-wide depletion of replication initiation events in highly transcribed regions. Genome Res 21: 1822–1832. 21813623 10.1101/gr.124644.111 PMC3205567

[47] Besnard E, Babled A, Lapasset L, Milhavet O, Parrinello H, et al (2012) Unraveling cell typespecific and reprogrammable human replication origin signatures associated with G-quadruplex consensus motifs. Nat Struct Mol Biol 19: 837–844. 22751019 10.1038/nsmb.2339

[48] Hamlin JL, Mesner LD, Dijkwel PA (2010) A winding road to origin discovery. Chromosome Res 18: 45–61. 19859818 10.1007/s10577-009-9089-z PMC2904547

[49] Valenzuela MS, Chen Y, Davis S, Yang F, Walker RL, et al (2011) Preferential localization of human origins of DNA replication at the 5′-ends of expressed genes and at evolutionarily conserved DNA sequences. PLoS One 6: e17308. 21602917 10.1371/journal.pone.0017308 PMC3094316

[50] Cayrou C, Coulombe P, Puy A, Rialle S, Kaplan N, et al (2012) New insights into replication origin characteristics in metazoans. Cell Cycle 11: 658–67. 22373526 10.4161/cc.11.4.19097 PMC3318102

[51] Raghuraman MK, Winzeler EA, Collingwood D, Hunt S, Wodicka L, et al (2001) Replication dynamics of the yeast genome. Science 294: 115–121. 11588253 10.1126/science.294.5540.115

[52] Lee TJ, Pascuzzi PE, Settlage SB, Shultz RW, Tanurdzic M, et al (2010) *Arabidopsis thaliana* chromosome 4 replicates in two phases that correlate with chromatin state. PLoS Genet 6: e1000982. 20548960 10.1371/journal.pgen.1000982 PMC2883604

[53] Schübeler D, Scalzo D, Kooperberg C, van Steensel B, Delrow J, et al (2002) Genome-wide DNA replication profile for *Drosophila melanogaster*: a link between transcription and replication timing. Nat Genet 32: 438–442. 12355067 10.1038/ng1005

[54] MacAlpine DM, Rodriguez HK, Bell SP (2004) Coordination of replication and transcription along a Drosophila chromosome. Genes Dev 18: 3094–3105. 15601823 10.1101/gad.1246404 PMC535919

[55] Farkash-Amar S, Lipson D, Polten A, Goren A, Helmstetter C, et al (2008) Global organization of replication time zones of the mouse genome. Genome Res 18: 1562–1570. 18669478 10.1101/gr.079566.108 PMC2556267

[56] Hiratani I, Ryba T, Itoh M, Yokochi T, Schwaiger M, et al (2008) Global reorganization of replication domains during embryonic stem cell differentiation. PLoS Biol 6: e245. 18842067 10.1371/journal.pbio.0060245 PMC2561079

[57] Hiratani I, Ryba T, Itoh M, Rathjen J, Kulik M, et al (2010) Genome-wide dynamics of replication timing revealed by in vitro models of mouse embryogenesis. Genome Res 20: 155–169. 19952138 10.1101/gr.099796.109 PMC2813472

[58] Woodfine K, Fiegler H, Beare DM, Collins JE, McCann OT, et al (2004) Replication timing of the human genome. Hum Mol Genet 13: 191–202. 14645202 10.1093/hmg/ddh016

[59] Desprat R, Thierry-Mieg D, Lailler N, Lajugie J, Schildkraut C, et al (2009) Predictable dynamic program of timing of DNA replication in human cells. Genome Res 19: 2288–2299. 19767418 10.1101/gr.094060.109 PMC2792175

[60] Chen CL, Rappailles A, Duquenne L, Huvet M, Guilbaud G, et al (2010) Impact of replication timing on non-CpG and CpG substitution rates in mammalian genomes. Genome Res 20: 447–457. 20103589 10.1101/gr.098947.109 PMC2847748

[61] Hansen RS, Thomas S, Sandstrom R, Canfield TK, Thurman RE, et al (2010) Sequencing newly replicated DNA reveals widespread plasticity in human replication timing. Proc Natl Acad Sci USA 107: 139–144. 19966280 10.1073/pnas.0912402107 PMC2806781

[62] Hiratani I, Takebayashi S, Lu J, Gilbert DM (2009) Replication timing and transcriptional control: beyond cause and effect part II. Curr Opin Genet Dev 19: 142–149. 19345088 10.1016/j.gde.2009.02.002 PMC2677117

[63] Schwaiger M, Stadler MB, Bell O, Kohler H, Oakeley EJ, et al (2009) Chromatin state marks cell-type- and gender-specific replication of the Drosophila genome. Genes Dev 23: 589–601. 19270159 10.1101/gad.511809 PMC2658520

[64] Farkash-Amar S, Simon I (2010) Genome-wide analysis of the replication program in mammals. Chromosome Res 18: 115–125. 20205353 10.1007/s10577-009-9091-5

[65] Ryba T, Hiratani I, Lu J, Itoh M, Kulik M, et al (2010) Evolutionarily conserved replication timing profiles predict long-range chromatin interactions and distinguish closely related cell types. Genome Res 20: 761–770. 20430782 10.1101/gr.099655.109 PMC2877573

[66] Zhou J, Ermakova OV, Riblet R, Birshtein BK, Schildkraut CL (2002) Replication and subnuclear location dynamics of the immunoglobulin heavy-chain locus in B-lineage cells. Mol Cell Biol 22: 4876–4889. 12052893 10.1128/MCB.22.13.4876-4889.2002 PMC133899

[67] Williams RR, Azuara V, Perry P, Sauer S, Dvorkina M, et al (2006) Neural induction promotes large-scale chromatin reorganisation of the Mash1 locus. J Cell Sci 119: 132–140. 16371653 10.1242/jcs.02727

[68] Takebayashi S, Dileep V, Ryba T, Dennis JH, Gilbert DM (2012) Chromatin-interaction compartment switch at developmentally regulated chromosomal domains reveals an unusual principle of chromatin folding. Proc Natl Acad Sci USA 109: 12574–12579. 22807480 10.1073/pnas.1207185109 PMC3411983

[69] Takebayashi S, Ryba T, Gilbert DM (2012) Developmental control of replication timing defines a new breed of chromosomal domains with a novel mechanism of chromatin unfolding. Nucleus 3: 500–507. 23023599 10.4161/nucl.22318 PMC3515532

[70] Zink D, Borneth H, Visser A, Cremer C, Cremer T (1999) Organization of early and late replicating DNA in human chromosome territories. Exp Cell Res 247: 176–188. 10047460 10.1006/excr.1998.4311

[71] Cook PR (1999) The organization of replication and transcription. Science 284: 1790–1795. 10364545 10.1126/science.284.5421.1790

[72] Berezney R (2002) Regulating the mammalian genome: the role of nuclear architecture. Adv Enzyme Regul 42: 39–52. 12123705 10.1016/s0065-2571(01)00041-3

[73] Grasser F, Neusser M, Fiegler H, Thormeyer T, Cremer M, et al (2008) Replication-timing-correlated spatial chromatin arrangements in cancer and in primate interphase nuclei. J Cell Sci 121: 1876–1886. 18477608 10.1242/jcs.026989 PMC2687722

[74] Lieberman-Aiden E, van Berkum NL, Williams L, Imakaev M, Ragoczy T, et al (2009) Comprehensive mapping of long-range interactions reveals folding principles of the human genome. Science 326: 289–293. 19815776 10.1126/science.1181369 PMC2858594

[75] Yaffe E, Farkash-Amar S, Polten A, Yakhini Z, Tanay A, et al (2010) Comparative analysis of DNA replication timing reveals conserved large-scale chromosomal architecture. PLoS Genet 6: e1001011. 20617169 10.1371/journal.pgen.1001011 PMC2895651

[76] Moindrot B, Audit B, Klous P, Baker A, Thermes C, et al (2012) 3D chromatin conformation correlates with replication timing and is conserved in resting cells. Nucleic Acids Res 40: 9470–9481. 22879376 10.1093/nar/gks736 PMC3479194

[77] Buongiorno-Nardelli M, Micheli G, Carri MT, Marilley M (1982) A relationship between replicon size and supercoiled loop domains in the eukaryotic genome. Nature 298: 100–102. 7088157 10.1038/298100a0

[78] Conti C, Sacca B, Herrick J, Lalou C, Pommier Y, et al (2007) Replication fork velocities at adjacent replication origins are coordinately modified during DNA replication in human cells. Mol Biol Cell 18: 3059–3067. 17522385 10.1091/mbc.E06-08-0689 PMC1949372

[79] Guilbaud G, Rappailles A, Baker A, Chen CL, Arneodo A, et al (2011) Evidence for sequential and increasing activation of replication origins along replication timing gradients in the human genome. PLoS Comput Biol 7: e1002322. 22219720 10.1371/journal.pcbi.1002322 PMC3248390

[80] Baker A, Audit B, Chen CL, Moindrot B, Leleu A, et al (2012) Replication fork polarity gradients revealed by megabase-sized U-shaped replication timing domains in human cell lines. PLoS Comput Biol 8: e1002443. 22496629 10.1371/journal.pcbi.1002443 PMC3320577

[81] Jackson DA, Pombo A (1998) Replicon clusters are stable units of chromosome structure: evidence that nuclear organization contributes to the efficient activation and propagation of S phase in human cells. J Cell Biol 140: 1285–1295. 9508763 10.1083/jcb.140.6.1285 PMC2132671

[82] Ma H, Samarabandu J, Devdhar RS, Acharya R, Cheng PC, et al (1998) Spatial and temporal dynamics of DNA replication sites in mammalian cells. J Cell Biol 143: 1415–1425. 9852140 10.1083/jcb.143.6.1415 PMC2132991

[83] Leonhardt H, Rahn HP, Weinzieri P, Sporbert A, Cremer T, et al (2000) Dynamics of DNA replication factories in living cells. J Cell Biol 149: 271–280. 10769021 10.1083/jcb.149.2.271 PMC2175147

[84] Cook PR (2001) Principles of Nuclear Structure and Functions. New York: Wiley.

[85] Carter DRF, Eskiw C, Cook PR (2008) Transcription factories. Biochem Soc Trans 36: 585–589. 18631121 10.1042/BST0360585

[86] Chambeyron S, Bickmore WA (2004) Does looping and clustering in the nucleus regulate gene expression? Curr Opin Cell Biol 16: 256–262. 15145349 10.1016/j.ceb.2004.03.004

[87] Izenman AJ (2008) Modern multivariate statistical techniques: regression, classification, and manifold learning. New York: Springer.

[88] Kaufman L, Rousseeuw PJ (1984) Finding groups in data: An introduction to cluster analysis. New York: John Wiley & Sons.

[89] Audit B, Baker A, Chen CL, Rappailles A, Guilbaud G, et al (2013) Multiscale analysis of genome-wide replication timing profiles using a wavelet-based signal-processing algorithm. Nat Protoc 8: 98–110. 23237832 10.1038/nprot.2012.145

[90] Brodie of Brodie EB, Nicolay S, Touchon M, Audit B, d'Aubenton-Carafa Y, et al (2005) From DNA sequence analysis to modeling replication in the human genome. Phys Rev Lett 94: 248103. 16090582 10.1103/PhysRevLett.94.248103

[91] Touchon M, Nicolay S, Audit B, Brodie of Brodie EB, d'Aubenton-Carafa Y, et al (2005) Replication-associated strand asymmetries in mammalian genomes: toward detection of replication origins. Proc Natl Acad Sci USA 102: 9836–9841. 15985556 10.1073/pnas.0500577102 PMC1174978

[92] Huvet M, Nicolay S, Touchon M, Audit B, d'Aubenton-Carafa Y, et al (2007) Human gene organization driven by the coordination of replication and transcription. Genome Res 17: 1278–1285. 17675363 10.1101/gr.6533407 PMC1950896

[93] Baker A, Nicolay S, Zaghloul L, d'Aubenton-Carafa Y, Thermes C, et al (2010) Wavelet-based method to disentangle transcription- and replication-associated strand asymmetries in mammalian genomes. Appl Comput Harmon Anal 28: 150–170.

[94] Chen CL, Duquenne L, Audit B, Guilbaud G, Rappailles A, et al (2011) Replication-associated mutational asymmetry in the human genome. Mol Biol Evol 28: 2327–2337. 21368316 10.1093/molbev/msr056

[95] Green P, Ewing B, Miller W, Thomas PJ, Green ED (2003) Transcription-associated mutational asymmetry in mammalian evolution. Nat Genet 33: 514–517. 12612582 10.1038/ng1103

[96] Touchon M, Nicolay S, Arneodo A, d'Aubenton-Carafa Y, Thermes C (2003) Transcription-coupled TA and GC strand asymmetries in the human genome. FEBS Lett 555: 579–582. 14675777 10.1016/s0014-5793(03)01306-1

[97] Touchon M, Arneodo A, d'Aubenton-Carafa Y, Thermes C (2004) Transcription-coupled and splicing-coupled strand asymmetries in eukaryotic genomes. Nucleic Acids Res 32: 4969–4978. 15388799 10.1093/nar/gkh823 PMC521644

[98] Baker A, Julienne H, Chen CL, Audit B, d'Aubenton Carafa Y, et al (2012) Linking the DNA strand asymmetry to the spatio-temporal replication program. I. About the role of the replication fork polarity in genome evolution. Eur Phys J E 35: 92. 23001787 10.1140/epje/i2012-12092-y

[99] Baker A, Chen CL, Julienne H, Audit B, d'Aubenton Carafa Y, et al (2012) Linking the DNA strand asymmetry to the spatio-temporal replication program: II. Accounting for neighbor-dependent substitution rates. Eur Phys J E 35: 123. 23179013 10.1140/epje/i2012-12123-9

[100] Audit B, Nicolay S, Huvet M, Touchon M, d'Aubenton Carafa Y, et al (2007) DNA replication timing data corroborate in silico human replication origin predictions. Phys Rev Lett 99: 248102. 18233493 10.1103/PhysRevLett.99.248102

[101] Audit B, Zaghloul L, Vaillant C, Chevereau G, d'Aubenton-Carafa Y, et al (2009) Open chromatin encoded in DNA sequence is the signature of “master” replication origins in human cells. Nucleic Acids Res 37: 6064–6075. 19671527 10.1093/nar/gkp631 PMC2764438

[102] Zaghloul L, Baker A, Audit B, Arneodo A (2012) Gene organization inside replication domains in mammalian genomes. C R Mécanique 340: 745–757.

[103] Lemaitre C, Zaghloul L, Sagot MF, Gautier C, Arneodo A, et al (2009) Analysis of fine-scale mammalian evolutionary breakpoints provides new insight into their relation to genome organisation. BMC Genomics 10: 335. 19630943 10.1186/1471-2164-10-335 PMC2722678

[104] Audit B, Zaghloul L, Baker A, Arneodo A, Chen CL, et al (2012) Megabase replication domains along the human genome: relation to chromatin structure and genome organisation. Subcell Biochem 61: 57–80. 10.1007/978-94-007-4525-4_3 23150246

[105] Barski A, Cuddapah S, Cui K, Roh TY, Schones DE, et al (2007) High-resolution profiling of histone methylations in the human genome. Cell 129: 823–837. 17512414 10.1016/j.cell.2007.05.009

[106] Chandra T, Kirschner K, Thuret JY, Pope BD, Ryba T, et al (2012) Independence of repressive histone marks and chromatin compaction during senescent heterochromatic layer formation. Mol Cell 47: 203–214. 22795131 10.1016/j.molcel.2012.06.010 PMC3701408

[107] Chessel D, Dufour A, Thioulouse J (2004) The ade4 package -I- One-table-methods. R News 4: 5–10.

[108] Rousseeuw P (1987) Silhouettes: a graphical aid to the interpretation and validation of cluster analysis. J Comput Appl Math 20: 53–65.

[109] Tibshirani R, Walther G, Hastie T (2001) Estimating the number of clusters in a data set via the gap statistic. J R Stat Soc: Series B (Stat Methodol) 63: 411–423.

[110] Minc E, Courvalin J, Buendia B (2000) Hp1gamma associates with euchromatin and heterochromatin in mammalian nuclei and chromosomes. Cytogenet Cell Genet 90: 279–284. 11124534 10.1159/000056789

[111] Li Y, Kirschmann DA, Wallrath LL (2002) Does heterochromatin protein 1 always follow code? Proc Natl Acad Sci USA 99 Suppl 4: 16462–16469. 12151603 10.1073/pnas.162371699 PMC139909

[112] Kellum R (2003) HP1 complexes and heterochromatin assembly. Curr Top Microbiol Immunol 274: 53–77. 12596904 10.1007/978-3-642-55747-7_3

[113] Maison C, Almouzni G (2004) HP1 and the dynamics of heterochromatin maintenance. Nat Rev Mol Cell Biol 5: 296–304. 15071554 10.1038/nrm1355

[114] Vakoc CR, Mandat SA, Olenchock BA, Blobel GA (2005) Histone H3 lysine 9 methylation and HP1*γ* are associated with transcription elongation through mammalian chromatin. Mol Cell 19: 381–391. 16061184 10.1016/j.molcel.2005.06.011

[115] Smallwood A, Hon GC, Jin F, Henry RE, Espinosa JM, et al (2012) CBX3 regulates efficient RNA processing genome-wide. Genome Res 22: 1426–1436. 22684280 10.1101/gr.124818.111 PMC3409256

[116] Kim TH, Abdullaev ZK, Smith AD, Ching KA, Loukinov DI, et al (2007) Analysis of the vertebrate insulator protein CTCF-binding sites in the human genome. Cell 128: 1231–1245. 17382889 10.1016/j.cell.2006.12.048 PMC2572726

[117] Phillips JE, Corces VG (2009) CTCF: master weaver of the genome. Cell 137: 1194–1211. 19563753 10.1016/j.cell.2009.06.001 PMC3040116

[118] Hon G, Wang W, Ren B (2009) Discovery and annotation of functional chromatin signatures in the human genome. PLoS Comput Biol 5: e1000566. 19918365 10.1371/journal.pcbi.1000566 PMC2775352

[119] Tardat M, Murr R, Herceg Z, Sardet C, Julien E (2007) PR-Set7-dependent lysine methylation ensures genome replication and stability through S phase. J Cell Biol 179: 1413–1426. 18158331 10.1083/jcb.200706179 PMC2373513

[120] Tardat M, Brustel J, Kirsh O, Lefevbre C, Callanan M, et al (2010) The histone H4 Lys 20 methyltransferase PR-Set7 regulates replication origins in mammalian cells. Nat Cell Biol 12: 1086–1093. 20953199 10.1038/ncb2113

[121] Brustel J, Tardat M, Kirsh O, Grimaud C, Julien E (2011) Coupling mitosis to DNA replication: the emerging role of the histone H4-lysine 20 methyltransferase PR-Set7. Trends Cell Biol 21: 452–460. 21632252 10.1016/j.tcb.2011.04.006

[122] Thurman RE, Day N, Noble WS, Stamatoyannopoulos JA (2007) Identification of higher-order functional domains in the human ENCODE regions. Genome Res 17: 917–927. 17568007 10.1101/gr.6081407 PMC1891350

[123] Škunca N, Altenhoff A, Dessimoz C (2012) Quality of computationally inferred gene ontology annotations. PLoS Comput Biology 8: e1002533. 10.1371/journal.pcbi.1002533 PMC3364937 22693439

[124] Klein E, Vánky F, Ben-Bassat H, Neumann H, Ralph P, et al (1976) Properties of the K562 cell line, derived from a patient with chronic myeloid leukemia. Int J Cancer 18: 421–431. 789258 10.1002/ijc.2910180405

[125] Drexler HG (2000) The Leukemia-Lymphoma Cell Line Factsbook. San Diego: Academic Press.

[126] Bernardi G (1995) The human genome: organization and evolutionary history. Annu Rev Genet 29: 445–476. 8825483 10.1146/annurev.ge.29.120195.002305

[127] Bernardi G (2001) Misunderstandings about isochores. Part 1. Gene 276: 3–13. 11591466 10.1016/s0378-1119(01)00644-8

[128] Eyre-Walker A, Hurst LD (2001) The evolution of isochores. Nat Rev Genet 2: 549–555. 11433361 10.1038/35080577

[129] Bird AP, Wolffe AP (1999) Methylation-induced repression–belts, braces, and chromatin. Cell 99: 451–454. 10589672 10.1016/s0092-8674(00)81532-9

[130] Suzuki MM, Bird A (2008) DNA methylation landscapes: provocative insights from epigenomics. Nat Rev Genet 9: 465–476. 18463664 10.1038/nrg2341

[131] Bird A (2002) DNA methylation patterns and epigenetic memory. Genes Dev 16: 6–21. 11782440 10.1101/gad.947102

[132] Goldar A, Marsolier-Kergoat MC, Hyrien O (2009) Universal temporal profile of replication origin activation in eukaryotes. PLoS One 4: e5899. 19521533 10.1371/journal.pone.0005899 PMC2690853

[133] Yang SCH, Rhind N, Bechhoefer J (2010) Modeling genome-wide replication kinetics reveals a mechanism for regulation of replication timing. Mol Syst Biol 6: 404. 20739926 10.1038/msb.2010.61 PMC2950085

[134] de Moura APS, Retkute R, Hawkins M, Nieduszynski CA (2010) Mathematical modelling of whole chromosome replication. Nucleic Acids Res 38: 5623–5633. 20457753 10.1093/nar/gkq343 PMC2943597

[135] Baker A, Audit B, Yang SCH, Bechhoefer J, Arneodo A (2012) Inferring where and when replication initiates from genome-wide replication timing data. Phys Rev Lett 108: 268101. 23005017 10.1103/PhysRevLett.108.268101

[136] Mortazavi A, Williams B, McCue K, Schaeffer L, Wold B (2008) Mapping and quantifying mammalian transcriptomes by RNA-seq. Nat Methods 5: 621–628. 18516045 10.1038/nmeth.1226 PMC13303166

[137] Benjamini Y, Yekutieli D (2001) The control of the false discovery rate in multiple testing under dependency. Ann Stat 29: 1165–1188.

